# Analysis of planes within reduced micromorphic model

**DOI:** 10.1038/s41598-021-94912-z

**Published:** 2021-07-30

**Authors:** A. R. El Dhaba, S. Mahmoud Mousavi

**Affiliations:** 1 Department of Mathematics, Faculty of Science, Damanhour University, Damanhour, Egypt; 2 Division of Applied Mechanics, Department of Materials Science and Engineering, Uppsala University, P.O. Box 534, 751 21 Uppsala, Sweden

**Keywords:** Mechanical engineering, Computational methods

## Abstract

A plane within reduced micromorphic model subjected to external static load is studied using the finite element method. The reduced micromorphic model is a generalized continuum theory which can be used to capture the interaction of the microstructure. In this approach, the microstructure is homogenized and replaced by a reduced micromorphic material model. Then, avoiding the complexity of the microstructure, the reduced micromorphic model is analyzed to reveal the interaction of the microstructure and the external loading. In this study, the three-dimensional formulation of the reduced micromorphic model is dimensionally reduced to address a plane under in-plane external load. The governing system of partial differential equations with corresponding consistent boundary conditions are discretized and solved using the finite element method. The classical and nonclassical deformation measures are then demonstrated and discussed for the first time for a material employing the reduced micromorphic model.

## Introduction

Materials with architectured microstructure provide efficient properties and have been in the focus of many research groups in the last few decades. Due to the feasibility of tailoring the microstructure to achieve auxetic behaviors, the analysis and design of such novel materials (referred also as metamaterials) are not possible within classical continuum mechanics. Accordingly, the generalized continua^1–4^ have regained the attention and have been employed for capturing the non-classical features of these materials such as size effect and band gap^5^. In addition to mechanical properties, such generalized theories have also been presented for multiphysics addressing e.g. electrical and magnetic properties^6^.

A thorough literature review on generalized continua is out of the scope and only a brief review is compiled here to introduce the subject. Nonlocal^7,8,23^, higher-order and higher-grade theories^9–11^ have been introduced as extensions of the classical continuum theories. Within higher order (or micro continuum theories), the classical three translational degrees of freedom (DOF) is augmented by additional DOFs stemming from the microstructure. These theories are two-level continuum models which consider the material as a collection of deformable point particles. Depending on the number of the additional DOFs, various theories such as micromorphic elasticity (with 12 DOFs), microstretch elasticity (with 7 DOFs), micropolar elasticity (with 6 DOFs)^12^, and dilatation elasticity (with 4 DOFs) are obtained.

An expected feature of the generalized continua is their numerous material constants which are to be determined for any specific microstructure via real or numerical experiments, e.g. see^13^. Although this task is commonly referred to as a “challenge” for the application of the generalized continua, one should not forget that capturing the nonclassical features are only possible due to the presence of such nonclassical material constants. Nevertheless, the generalization of the continuum theory should be performed with care to employ the most efficient model with the minimum number of material constants for the microstructure and the objective physical phenomenon to be captured. For this purpose, recently, computational homogenization has been successfully used for identifying and realizing a suitable continuum theory^14–20,38^. To this end, a comprehensive methodology capable of identifying the most efficient continuum theory for a specific heterogeneous material is not yet identified.

In addition to more classical versions of generalized continua, recent developments have been reported in the literature such as relaxed micromorphic model^21^ and reduced micromorphic model^22^. These models aim for filling the gaps in the realm of the generalized continua to provide an efficient selection of a generalized model without the need to include unnecessary extensions. In an attempt to reduce the number of material parameters and degrees of freedom, Neff et al*.*^21^ proposed a relaxed micromorphic model which uses the curl of the micro-deformation tensor as a micro-dislocation measure. Furthermore, the coupling between the micro-strain and the macro-strain is eliminated in this model. The relaxed micromorphic model has been used to model the wave propagation in metamaterials^24–27^.

Another form of the micromorphic model is proposed by Shaat^22^ which is referred to as the reduced micromorphic model (referred to, in this text, as RMM). In this model the author considers the coupling between the micro-strain and the macro-strain to measure the concept of so-called residual strain and consequently the residual stress. The model has been employed to investigate the wave propagation in metamaterials and composite materials. An application for the model is also introduced in^28^ to define the equivalent shear modulus of composite metamaterials. The reduced micromorphic model is also formulated in orthogonal curvilinear coordinates and an application to a metamaterial hemisphere has been reported using spherical coordinates^29^. Later, Shaat et al*.* introduced a micromorphic beam theory^30^ based on the reduced micromorphic model.

In the absence or due the complexity of an analytical solution^31^, the finite element method is an efficient numerical treatment of continuum theories. In this paper, the reduced micromorphic model is used together with the finite element method for the analysis of a plane. The framework is dimensionally reduced for planar analysis. Such dimension reduction is motivated as it can efficiently be used for the analysis of 2D structures^32^. The corresponding measures of deformation are elaborated to demonstrate the response of a plane within RMM subjected to static in-plane loading.

The paper is organized as follows. The reduced micromorphic model is reviewed in Section “The RMM in Cartesian coordinates”. In Section “The RMM model in 2-Dimension”, the proposed domain in two dimensions and the planar formulation are presented. After introducing the governing field equations and the variationally consistent boundary conditions, two case studies are defined. In Section “Numerical results”. the finite element analysis and numerical simulation of the two case studies are reported. Finally, the conclusion is given in Section “Conclusion”.

## The RMM in Cartesian coordinates

### The kinematical variables

The classical theories of continuum mechanics do not have the ability to represent the nanoscale phenomena. Accordingly, the generalized continua such as the reduced micromorphic model (RMM) is introduced as an alternative for studying such phenomena at the micro-scale level, where the model introduces the micro-strain tensor as an additional measure besides the displacement field. Moreover, it introduces the coupling between the strain tensor and the micro-strain tensor as a coupling measure with the elimination of the repeated effects.

In addition to its ability to reducing the material parameters, the RMM generates additional field equations and reduces the order of the partial differential equations of the model. Such reduction sometimes facilitates obtaining the analytical solutions.

The deformation occurring for elastic materials is an accumulative movement for the material points at the nanoscale. Moreover, the tools used in classical theories to measure the displacement ignore the internal movement of such points and is only limited to the movement of the surface points. Consequently, introducing new measures to describe the internal movement of the material points and eliminating the repeated effects resultant from two different actions, the kinematics of RMM is given by

1 $$\varepsilon_{ij} = \frac{1}{2}\left( {u_{i,j} + u_{j,i} } \right),\quad \gamma_{ij} = \varepsilon_{ij} - s_{ij} ,\quad \chi_{ijk} = s_{jk,i} ,\quad \chi_{ijj} = 0,$$

where $\varepsilon_{ij} \left( { = \varepsilon_{ji} } \right)$ denote the classical strain tensor, $s_{ij} \left( { = s_{ji} } \right)$ is the micro-strain tensor, $\gamma_{ij} \left( { = \gamma_{ji} } \right)$ is the coupling between the micro-strain $s_{ij}$ and the macro-strain $\varepsilon_{ij}$ and $\chi_{ijk} \left( { = \chi_{ikj} } \right)$ is the gradient of the micro-strain tensor $s_{ij}$.

### Equations of motion

The variational method is a mathematical procedure that is used to obtain the field equations and the corresponding boundary conditions for the considered model. This procedure is used widely in continuum mechanics, fluid mechanics, optics, quantum mechanics, thermodynamics and electromagnetism. The total free energy function for the volume $\Omega$(volume of the body) bounded by the surface $\partial\Omega$(surface of the body) is considered as a function with internal variables as

2 $$W=\underset{\Omega }{\overset{}{\int }}\overline{W }\left({s}_{ij}, {\gamma }_{ij}, {s}_{ij,k}\right) dV.$$

The first variation for the total free energy reads

3 $$\delta W=\underset{\Omega }{\overset{}{\int }}\left(\frac{\partial \overline{W}}{\partial {s }_{ij}}\delta {s}_{ij}+\frac{\partial \overline{W}}{\partial {\gamma }_{ij}}\delta {\gamma }_{ij}+\frac{\partial \overline{W}}{\partial {s }_{ij,k}}\delta {s}_{ij,k}\right)dV.$$

The constitutive relations are defined as follows

4 $${t}_{ij}=\frac{\partial \overline{W}}{\partial {s }_{ij}},\quad {\tau }_{ij}=\frac{\partial \overline{W}}{\partial {\gamma }_{ij}},\quad {m}_{ijk}=\frac{\partial \overline{W}}{\partial {s }_{jk,i}},$$

where ${t}_{ij}$ is the micro-stress tensor, ${\tau }_{ij}$ is a Cauchy-like tensor or residual stress tensor, and ${m}_{ijk}$ is the higher order stress tensor, respectively.

Substituting Eq. (4) into Eq. (3) results in

5 $$\delta W=\underset{\Omega }{\overset{}{\int }}\left({t}_{ij} \delta {s}_{ij}+{\tau }_{ij} \delta {\gamma }_{ij}+{m}_{kij} \delta {s}_{ij,k}\right)dV.$$

Similar to the free energy, the kinetic energy is also generalized by considering the micro-inertia energy as

6 $$K = \frac{1}{2}\mathop \int \limits_{{\Omega }}^{{}} \left( {\rho \dot{u}_{i}^{2} + \rho_{m} J\dot{s}_{jk}^{2} } \right)dV,$$

where $\rho$ is the macroscopic mass density of the metamaterial, $\rho_{m}$ is the mass density of the material particle, and $J$ denotes its micro-inertia. Additional terms can be considered in the kinetic energy to describe complex phenomena in the metamaterials as in^33^.

The first variation of kinetic energy is

7 $$\delta K = \int\limits_{\Omega }^{{}} {\left( {\rho \mathop {\ddot{u}}\nolimits_{i} \delta u_{i} + \rho _{m} J\mathop {\ddot{s}}\nolimits_{{jk}} \delta s_{{jk}} } \right)} dV.$$

Therefore, the total variation for internal energy reads

8 $$\delta W + \delta K = \int\limits_{\Omega }^{{}} {\left( {t_{{ij}} \delta s_{{ij}} + \tau _{{ij}} \delta \gamma _{{ij}} + m_{{kij}} \delta s_{{ij,k}} } \right)} dV + \int\limits_{\Omega }^{{}} {\left( {\rho \mathop {\ddot{u}}\nolimits_{i} \delta u_{i} + \rho _{m} J\mathop {\ddot{s}}\nolimits_{{jk}} \delta s_{{jk}} } \right)} dV.$$

The work done by the external forces, $\tilde{W}$, is defined as

9 $$\tilde{W} = \mathop \int \limits_{\Omega }^{{}} \left( {f_{i} u_{i} + H_{jk} s_{jk} } \right) dV + \mathop \int \limits_{\partial \Omega }^{{}} \left( {\overline{t}_{i} u_{i} + \overline{m}_{jk} s_{jk} } \right) dS + \mathop \int \limits_{\partial \partial \Omega }^{{}} F_{i}^{ex} u_{i} dL,$$

where ${f}_{i}$ is the body force, ${H}_{jk}$ is the body higher-order moment, ${\overline{t} }_{i}$ is the Cauchy stress vector, ${\overline{m} }_{jk}$ is the higher stress tensor or double force, and $F_{i}^{ex}$ is the wedge forces at the corners of the domain^34^. Here, $\partial \partial {\Omega }$ are the vertex points. The variation of external work reads

10 $$\delta \tilde{W} = \mathop \int \limits_{\Omega }^{{}} \left( {f_{i} \delta u_{i} + H_{jk} \delta s_{jk} } \right)dV + \mathop \int \limits_{\partial \Omega }^{{}} \left( {\overline{t}_{i} \delta u_{i} + \overline{m}_{jk} \delta s_{jk} } \right)dS + \mathop \int \limits_{\partial \partial \Omega }^{{}} F_{i}^{ex} \delta u_{i} dL.$$

Considering Eqs. (8) and (10), the Hamilton’s principle results in

11 $$\begin{aligned}& \underset{\Omega }{\overset{}{\int }}\left({t}_{ij} \delta {s}_{ij}+{\tau }_{ij} \delta {\gamma }_{ij}+{m}_{kij} \delta {s}_{ij,k}\right)dV+{\int }_{\Omega }\left(\rho {\ddot{u}}_{i}\delta {u}_{i}+{\rho }_{m}J{\ddot{s}}_{jk}\updelta {s}_{jk}\right)dV\\ &\quad=\underset{\Omega }{\overset{}{\int }}\left({f}_{i}\delta {u}_{i}+{H}_{jk}\delta {s}_{jk}\right)dV+\underset{\partial \Omega }{\overset{}{\int }}\left({\overline{t} }_{i}\delta {u}_{i}+{\overline{m} }_{jk}\delta {s}_{jk}\right)dS+\underset{\partial \partial \Omega }{\overset{}{\int }}{F}_{i}^{ex}\delta {u}_{i}dL.\end{aligned}$$

Applying the divergence theorem, we get

12 $$ \begin{aligned} & \int\limits_{\Omega }^{{}} {\left( {\left( {t_{{ij}} - \tau _{{ij}} - m_{{kij,k}} - H_{{ij}} + \rho _{m} Js_{{ij}} } \right)\delta s_{{ij}} + \left( {\rho {\text{ }}u_{i} - \tau _{{ij,j}} - f_{i} } \right)\delta u_{i} } \right)} dV \\&\quad + \int\limits_{{\partial \Omega }}^{{}} {\left( {\left( {n_{j} \tau _{{ij}} - \bar{t}_{i} } \right)\delta \dot{u}_{i} + \left( {n_{k} m_{{kij}} - \bar{m}_{{ij}} } \right)\delta \dot{s}_{{ij}} } \right)} dS - \int\limits_{{\partial \partial \Omega }}^{{}} {F_{i}^{{ex}} } \delta u_{i} dL.{\text{ }}\end{aligned} $$

Equation (12) is satisfied for all volumes bounded by smooth and unsmooth boundaries if and only if the integrands are zero. Consequently, equations of motion and corresponding boundary conditions for smooth boundaries enclosed a bounded volume are

13 $$\tau_{ji,j} + f_{i} = \rho \mathop {\ddot{u}}\nolimits_{i} , m_{ijk,i} + \tau_{jk} - t_{jk} + H_{jk} = \rho_{m} J\mathop {\ddot{s}}\nolimits_{{jk}} ,$$

and

14 $$n_{j} \tau_{ji} = \overline{{t_{i} }} ,\quad n_{i} m_{ijk} = \overline{m}_{jk}.$$

### Internal energy

According to^22,28–30^, the free energy in RMM is taken in the form

15 $$W = \frac{1}{2}\lambda_{m} s_{ii} s_{jj} + \mu_{m} s_{ij} s_{ij} + \frac{1}{2}\lambda \gamma_{ii} \gamma_{jj} + \mu \gamma_{ij} \gamma_{ij} + \lambda_{c} \gamma_{ii} s_{jj} + 2\mu_{c} \gamma_{ij} s_{ij} + \frac{1}{2}\lambda_{m} \ell_{1}^{2} \left( {\chi_{iik} \chi_{jjk} + \chi_{ijk} \chi_{jik} } \right) + \frac{1}{2}\mu_{m} \ell_{2}^{2} \chi_{ijk} \chi_{ijk} ,$$

where ${\lambda }_{m}$ and ${\mu }_{m}$ are the elastic moduli of the microstructure, $\lambda$ and $\mu$ are the elastic moduli of the matrix material between two particles, ${\lambda }_{c}$ and ${\mu }_{c}$ are two elastic moduli accounting for the coupling between the micro-strain and the macro-strain, and ${{\ell}}_{1}$ and ${{\ell}}_{2}$ are length scale parameters. The free energy function in two dimensions can be written in a matrix form as^35^

16 $$ \begin{aligned}W &= \left[ {\begin{array}{*{20}c} {s_{{11}} } & {s_{{22}} } & {s_{{12}} } \\ \end{array} } \right]\left[ {\begin{array}{*{20}c} {\frac{1}{2}\lambda _{m} + \mu _{m} } & {\frac{1}{2}\lambda _{m} } & 0 \\ {\frac{1}{2}\lambda _{m} } & {\frac{1}{2}\lambda _{m} + \mu _{m} } & 0 \\ 0 & 0 & {2\mu _{m} } \\ \end{array} } \right]\left[ {\begin{array}{*{20}c} {s_{{11}} } \\ {s_{{22}} } \\ {s_{{12}} } \\ \end{array} } \right] + \left[ {\begin{array}{*{20}c} {\gamma _{{11}} } & {\gamma _{{22}} } & {\gamma _{{12}} } \\ \end{array} } \right]\left[ {\begin{array}{*{20}c} {\frac{1}{2}\lambda + \mu } & {\frac{1}{2}\lambda + \mu } & 0 \\ {\frac{1}{2}\lambda + \mu } & {\frac{1}{2}\lambda + \mu } & 0 \\ 0 & 0 & 0 \\ \end{array} } \right]\left[ {\begin{array}{*{20}c} {\gamma _{{11}} } \\ {\gamma _{{22}} } \\ {\gamma _{{12}} } \\ \end{array} } \right]\\&\quad + \left[ {\begin{array}{*{20}c} {s_{{11}} } & {s_{{22}} } & {s_{{12}} } \\ \end{array} } \right]\left[ {\begin{array}{*{20}c} {\lambda _{c} + 2\mu _{c} } & {\lambda _{c} } & 0 \\ {\lambda _{c} } & {\lambda _{c} + 2\mu _{c} } & 0 \\ 0 & 0 & {4\mu _{c} } \\ \end{array} } \right]\left[ {\begin{array}{*{20}c} {\gamma _{{11}} } \\ {\gamma _{{22}} } \\ {\gamma _{{12}} } \\ \end{array} } \right] + \left[ {\begin{array}{*{20}c} {\chi _{{112}} } & {\chi _{{221}} } \\ \end{array} } \right]\left[ {\begin{array}{*{20}c} {\lambda _{m} \ell_{1}^{2} + \mu _{m} \ell_{2}^{2} } & 0 \\ 0 & {\lambda _{m} \ell_{1}^{2} + \mu _{m} \ell_{2}^{2} } \\ \end{array} } \right]\left[ {\begin{array}{*{20}c} {\chi _{{112}} } \\ {\chi _{{221}} } \\ \end{array} } \right].\end{aligned} $$

Therefore, the function $W$ is positive definite if the following conditions are satisfied

$$\begin{array}{ccc}{{\lambda}}_{{m}}+2{{\mu}}_{{m}}\ge 0,& {\mu }_{m}\ge 0,& {{\lambda}}_{{m}}+{{\mu}}_{{m}}\ge 0,\\ & & \\ {{\lambda}}_{{c}}+2{{\mu}}_{{c}}\ge 0,& {\mu }_{c}\ge 0,& {{\lambda}}_{{c}}+{{\mu}}_{{c}}\ge 0,\\ & & \\ \lambda +2\mu \ge 0,& \mu \ge 0,& \lambda +\lambda \ge 0.\end{array}$$

### The constitutive relations

The constitutive relations are related to the free energy function by

17 $${t}_{ij}=\frac{\partial W}{\partial {s}_{ij}},\quad \hspace{1em}{\tau }_{ij}=\frac{\partial W}{\partial {\gamma }_{ij}},\quad \hspace{1em}{m}_{ijk}=\frac{\partial W}{\partial {\chi }_{ijk}}.$$

Substituting the Eq. (15) into Eq. (17) and considering the deformation measures (1), the stress measures can be expressed in terms of deformation measures as

18 $$\begin{array}{*{20}c} {t_{{ij}} = \left( {\lambda - 2\lambda _{c} + \lambda _{m} } \right)\delta _{{ij}} s_{{qq}} + 2\left( {\mu - 2\mu _{c} + \mu _{m} } \right)s_{{ij}} - \left( {\lambda - \lambda _{c} } \right)\delta _{{ij}} u_{{q,q}} - \left( {{\upmu } - {\upmu }_{{\text{c}}} } \right)\left( {u_{{i,j}} + u_{{j,i}} } \right),} \\ {} \\ {\tau _{{ij}} = \left( {\lambda - \lambda _{c} } \right)\delta _{{ij}} u_{{q,q}} + \left( {\mu - \mu _{{\text{c}}} } \right)\left( {u_{{i,j}} + u_{{j,i}} } \right) - 2\left( {\mu - 2\mu _{c} } \right)s_{{ij}} - \left( {\lambda - 2\lambda _{c} } \right)\delta _{{ij}} s_{{qq}} ,} \\ {} \\ {m_{{ijk}} = \frac{1}{2}\lambda _{m} \ell_{1}^{2} \left( {\delta _{{ij}} s_{{qk,q}} + \delta _{{ik}} s_{{qj,q}} + s_{{ik,j}} + s_{{ij,k}} } \right) + \mu _{m} \ell_{2}^{2} s_{{jk,i}} .} \\ \end{array}$$

### General field equations

Considering the Eqs. (13) and (18) and using the condition $\chi_{ijj = 0}$, the general field equations read

19 $$\left( {\mu - \mu _{c} } \right)\nabla ^{2} u_{i} + \left( {\lambda - \lambda _{{\text{c}}} + \mu - \mu _{c} } \right)u_{{j,ji}} - 2\left( {\mu - 2\mu _{c} } \right)s_{{ij,j}} + f_{i} = \rho \mathop {\ddot{u}}\nolimits_{i} ,$$

20 $$\mu_{m} \ell_{2}^{2} \nabla^{2} s_{jk} + \frac{1}{2}\lambda_{m} \ell_{1}^{2} \left( {s_{mk,mj} + s_{mj,mk} + s_{ik,ij} + s_{ij,ik} } \right) - \left( {2\lambda - 4\lambda_{c} + \lambda_{m} } \right)\delta_{kj} s_{qq} - 2\left( {2\mu - 4\mu_{c} + \mu_{m} } \right)s_{kj} + 2\left( {\lambda - {\uplambda }_{{\text{c}}} } \right)\delta_{kj} u_{q,q} + 2\left( {\mu - \mu_{c} } \right)\left( {u_{k,j} + u_{j,k} } \right) + H_{jk} = \rho_{m} J\mathop {\ddot{s}}\nolimits_{{jk}} .$$

Equations (19), (20) represent the governing field equations for the RMM model, which should be satisfied inside the domain of the solution. In the limiting case, by neglecting the micro-strain tensors and the second equation, the RRM model is reduced to the classical model of elasticity.

## The RMM model in 2-Dimension

### The field equations in 2D

We consider a rectangular ($a \times b$) plane which behaves according to the RMM model. In order to study the interaction between the applied forces and the induced internal fields in the material, we consider planar displacement field and micro-strain field as follows:

21 $$u_{i} = u_{i} \left( {x,y} \right),\quad s_{ij} = s_{ij} \left( {x,y} \right),\quad i,j = x,y.$$

According to the reduced dimensions of the problem, the non-vanishing components of kinematic relations are

22 $$\begin{array}{*{20}c} {\varepsilon_{xx} = \frac{{\partial u_{x} }}{\partial x}, } & {\varepsilon_{yy} = \frac{{\partial u_{y} }}{\partial y}, } & {\varepsilon_{xy} = \frac{1}{2}\left( {\frac{{\partial u_{x} }}{\partial y} + \frac{{\partial u_{y} }}{\partial x}} \right), } \\ {\gamma_{xx} = \frac{{\partial u_{x} }}{\partial x} - s_{xx} }, & {\gamma_{yy} = \frac{{\partial u_{y} }}{\partial y} - s_{yy} ,} & {\gamma_{xy} = \frac{1}{2}\left( {\frac{{\partial u_{x} }}{\partial y} + \frac{{\partial u_{y} }}{\partial x}} \right) - s_{xy} ,} \\ \end{array}$$

while the following quantities are eliminated

$$\frac{{s_{xx} }}{\partial y} = \frac{{s_{yy} }}{\partial x} = 0.$$

The components of the micro-stress tensor (Eq. (18)_1_) take the form

$$t_{xx} = \left( {A_{1} + A_{2} + A_{5} } \right)s_{xx} + \left( {A_{3} + A_{6} } \right)s_{yy} - \left( {2A_{0} + A_{6} } \right)\frac{{\partial u_{x} }}{\partial x} - A_{6} \frac{{\partial u_{y} }}{\partial y},$$

23 $$t_{yy} = \left( {A_{3} + A_{6} } \right)s_{xx} + \left( {A_{1} + A_{2} + A_{5} } \right)s_{yy} - A_{6} \frac{{\partial u_{x} }}{\partial x} - \left( {2A_{0} + A_{6} } \right)\frac{{\partial u_{y} }}{\partial y},$$

$$t_{xy} = A_{4} s_{xy} - A_{0} \left( {\frac{{\partial u_{x} }}{\partial y} + \frac{{\partial u_{y} }}{\partial x}} \right).$$

The components of the so-called residual stress tensor (Eq. (18)_2_) read

24 $$\begin{aligned} \tau_{xx} & = \left( {2A_{0} + A_{6} } \right)\frac{{\partial u_{x} }}{\partial x} + A_{6} \frac{{\partial u_{y} }}{\partial y} - \left( {A_{1} + A_{5} } \right)s_{xx} - A_{1} s_{yy} , \\ \tau_{yy} & = A_{6} \frac{{\partial u_{x} }}{\partial x} + \left( {2A_{0} + A_{6} } \right)\frac{{\partial u_{y} }}{\partial y} - A_{1} s_{xx} - \left( {A_{1} + A_{5} } \right)s_{yy} , \\ \tau_{xy} & = A_{0} \left( {\frac{{\partial u_{x} }}{\partial y} + \frac{{\partial u_{y} }}{\partial x}} \right) - A_{5} s_{xy} . \\ \end{aligned}$$

Finally. the components of higher order micro-stress tensor (Eq. (18)_3_) are

25 $$\begin{array}{*{20}c} {m_{xxx} = m_{xyy} = \frac{1}{2}A_{7} \frac{{\partial s_{xy} }}{\partial y},\quad m_{yyy} = m_{yxx} = \frac{1}{2}A_{7} \frac{{\partial s_{xy} }}{\partial x}, } \\ {m_{yyx} = A_{8} \frac{{\partial s_{xy} }}{\partial y},\quad m_{xxy} = A_{8} \frac{{\partial s_{xy} }}{\partial x},} \\ \end{array}$$

with

$$\begin{array}{*{20}c} {A_{0} = \mu - \mu_{c} }, & {A_{1} = \lambda - 2\lambda_{c} }, & {A_{2} = \lambda_{m} + 2\mu_{m} }, & {A_{3} = \lambda_{m} - \lambda_{c} }, \\ {A_{4} = 2\left( {\mu - 2\mu_{c} + \mu_{m} } \right)}, & {A_{5} = 2\left( {\mu - 2\mu_{c} } \right)}, & {A_{6} = \lambda - {\uplambda }_{{\text{c}}} }, & {A_{7} = 2\lambda_{m} \ell_{1}^{2} }, \\ {A_{8} = \lambda_{m} \ell_{1}^{2} + \mu_{m} \ell_{2}^{2} }, & {A_{9} = 2A_{6} + A_{3} - {\uplambda }_{{\text{c}}} }, & {} & {} \\ \end{array}$$

$$\begin{array}{*{20}c} {A_{10} = 4A_{0} + A_{2} + A_{3} + 2A_{6} - \lambda_{m} - {\uplambda }_{{\text{c}}} - 4\mu_{c} }, & {A_{1} + \lambda_{m} = A_{3} + A_{6} }. & {} \\ \end{array}$$

In the static equilibrium and in the absence of body forces and body higher-order-moments, equations of motion (19)–(20) are given in expanded form as

26 $$A_{0} \nabla^{2} u_{x} + \left( {A_{0} + A_{6} } \right)\frac{\partial }{\partial x}\left( {\frac{{\partial u_{x} }}{\partial x} + \frac{{\partial u_{y} }}{\partial y}} \right) - A_{5} \frac{{\partial s_{xy} }}{\partial y} = 0,$$

27 $$A_{0} \nabla^{2} u_{y} + \left( {A_{0} + A_{6} } \right)\frac{\partial }{\partial y}\left( {\frac{{\partial u_{x} }}{\partial x} + \frac{{\partial u_{y} }}{\partial y}} \right) - A_{5} \frac{{\partial s_{xy} }}{\partial x} = 0,$$

28 $$A_{7} \frac{{\partial^{2} s_{xy} }}{\partial x\partial y} + 2\left( {2A_{0} + A_{6} } \right)\frac{{\partial u_{x} }}{\partial x} + 2A_{6} \frac{{\partial u_{y} }}{\partial y} - A_{10} s_{xx} - A_{9} s_{yy} = 0,$$

29 $$A_{7} \frac{{\partial^{2} s_{xy} }}{\partial x\partial y} + 2A_{6} \frac{{\partial u_{x} }}{\partial x} + 2\left( {2A_{0} + A_{6} } \right)\frac{{\partial u_{y} }}{\partial y} - A_{9} s_{xx} - A_{10} s_{yy} = 0,$$

30 $$A_{8} \nabla^{2} s_{xy} - \left( {A_{10} - A_{9} } \right)s_{xy} + 2A_{0} \left( {\frac{{\partial u_{x} }}{\partial y} + \frac{{\partial u_{y} }}{\partial x}} \right) = 0.$$

### The boundary conditions

To get a solution for the coupled system of partial differential Eqs. (26–30), we consider two boundary value problems where the domain of the solution is a rectangle with width $a$ and height $b$. The first boundary value problem (BVP) and the second BVP are presented graphically in Figs. 2, 3.

**Figure 1:**
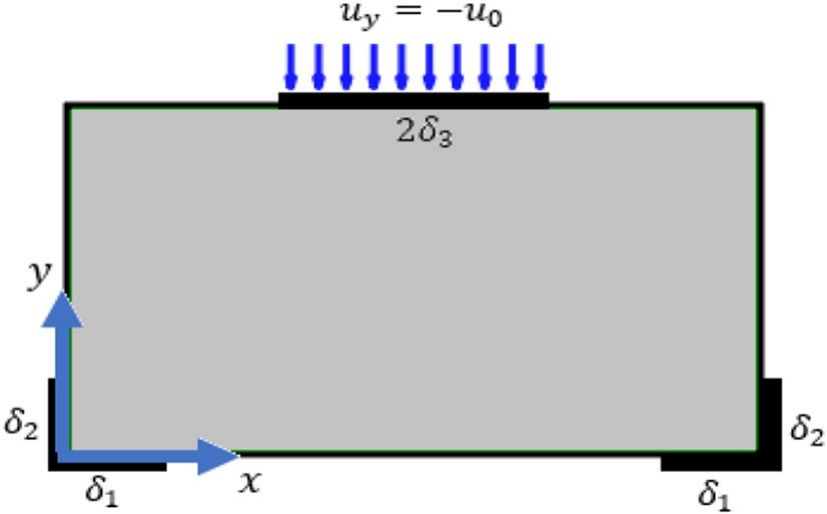
First boundary value problem.

**Figure 2:**
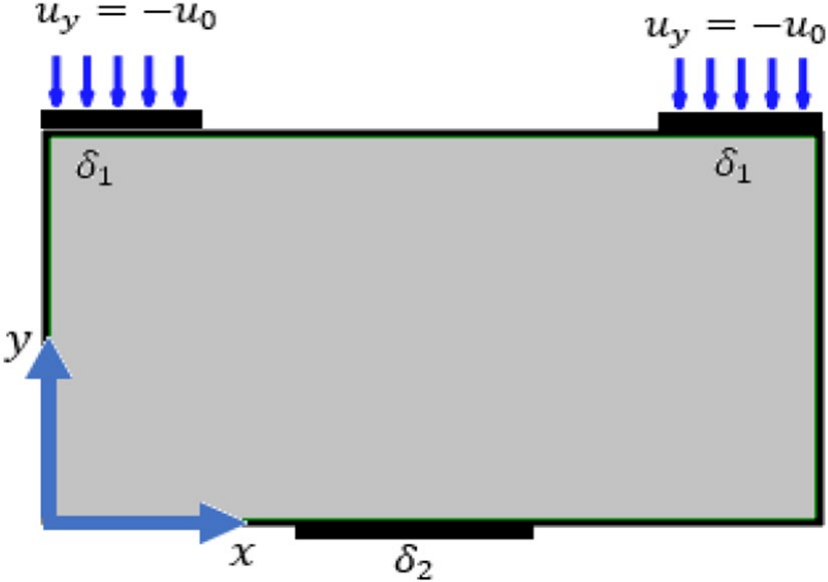
Second boundary value problem.

The mathematical expressions for the boundary conditions in Figs. 1, 2 are given here demonstrating the corresponding Dirichlet boundary conditions. Note that on the other boundaries, the Neumann boundary condition is applied being zero external tractions ($\overline{{t}_{i}}, {\overline{m}}_{jk}$).

1) Boundary conditions for the first boundary value problem:

31 $$\begin{array}{cccc}{u}_{x}={u}_{y}={s}_{xx}={s}_{yy}={s}_{xy}=0,& \forall & 0\le x\le {\delta }_{1},& y=0,\\ {u}_{x}={u}_{y}={s}_{xx}={s}_{yy}={s}_{xy}=0,& \forall & a-{\delta }_{1}\le x\le a,& y=0,\\ {u}_{x}={u}_{y}={s}_{xx}={s}_{yy}={s}_{xy}=0,& \forall & x=0,& 0\le y\le {\delta }_{2},\\ {u}_{x}={u}_{y}={s}_{xx}={s}_{yy}={s}_{xy}=0,& \forall & x=a,& 0\le y\le {\delta }_{2},\\ {u}_{y}=- {u}_{0}, {u}_{x}={s}_{xx}={s}_{yy}={s}_{xy}=0,& \forall & \frac{a}{2}-{\delta }_{3}\le x\le \frac{a}{2}+{\delta }_{3},& y=b.\end{array}$$

2) Boundary conditions for the second boundary value problem:

32 $$\begin{array}{*{20}c} {u_{x} = u_{y} = s_{xx} = s_{yy} = s_{xy} = 0,} & \forall & {\frac{a}{2} - \frac{{\delta_{2} }}{2} \le x \le \frac{a}{2} + \frac{{\delta_{2} }}{2},} & {y = 0,} \\ {u_{y} = - u_{0} , u_{x} = s_{xx} = s_{yy} = s_{xy} = 0,} & \forall & {0 \le x \le \delta_{1} ,} & {y = b,} \\ {u_{y} = - u_{0} , u_{x} = s_{xx} = s_{yy} = s_{xy} = 0,} & \forall & {a - \delta_{1} \le x \le a,} & {y = b.} \\ \end{array}$$

## Numerical results

The boundary value problem, i.e. Equations (26–30) with the corresponding boundary conditions (31) and (32), are numerically solved using the finite element method. For this purpose, the COMSOL Multiphysics^36,37^ is used for the numerical treatment of the boundary value problem. It is noted that, similar to the other generalized continua, the RMM is not available in the finite element software such as COMSOL Multiphysics, and the boundary value problem should be implemented to use the software as a solver to the system of partial differential equations. In the absence of experimental data, the behavior of the solution and the results are interpreted qualitatively.

### The material constants

The material constants given in the Table 1 are used for the analysis of the plane. A more realistic input values necessities the homogenization of the heterogeneous microstructure^38^.

**Table 1:** The material properties.

Dimension of square	$a = 0.1 m$*,* $b = 0.04 m$
Density	$\rho = 2700 N/m^{2}$
Lame’s coefficients	$\lambda = 5.1 \times 10^{10} N/m^{2}$*,* $\mu = 2.6 \times 10^{10} N/m^{2}$
Lame’s coefficients	$\lambda_{m} = 1.79 \times 10^{11} N/m^{2}$*,* $\mu_{m} = 1.41 \times 10^{11} N/m^{2}$
Length scales	$\ell_{1} = \ell_{2} = 0, 0.001, 0.004, 0.008 m,$
The rule of mixture	$\lambda_{e} = f\lambda_{m} + \left( {1 - f} \right)\lambda , \mu_{e} = f\mu_{m} + \left( {1 - f} \right)\mu ,$
Coupling moduli	$\lambda_{c} = 0, \mu_{c} = 0.$

In order to implement the system of Eqs. (26–30), they are expressed in the following matrix form

33 $$-c{\nabla }^{2}X+aX=f,$$

where

$$c=\left(\begin{array}{ccccc}{A}_{0}& 0& 0& 0& 0\\ 0& {A}_{0}& 0& 0& 0\\ 0& 0& 0& 0& 0\\ 0& 0& 0& 0& 0\\ 0& 0& 0& 0& {A}_{8}\end{array}\right),\quad a=\left(\begin{array}{ccccc}0& 0& 0& 0& 0\\ 0& 0& 0& 0& 0\\ 0& 0& -{A}_{10}& -{A}_{9}& 0\\ 0& 0& -{A}_{9}& -{A}_{10}& 0\\ 0& 0& 0& 0& -\left({A}_{10}-{A}_{9}\right)\end{array}\right), \quad X=\left(\begin{array}{c}{{\varvec{u}}}_{{\varvec{x}}}\\ {{\varvec{u}}}_{{\varvec{y}}}\\ {{\varvec{s}}}_{{\varvec{x}}{\varvec{x}}}\\ {{\varvec{s}}}_{{\varvec{y}}{\varvec{y}}}\\ {{\varvec{s}}}_{{\varvec{x}}{\varvec{y}}}\end{array}\right),$$

$$f=\left(\begin{array}{c}\left({A}_{0}+{A}_{6}\right)\frac{\partial }{\partial x}\left(\frac{\partial {u}_{x}}{\partial x}+\frac{\partial {u}_{y}}{\partial y}\right)-{A}_{5}\frac{\partial {s}_{xy}}{\partial y}\\ \left({A}_{0}+{A}_{6}\right)\frac{\partial }{\partial y}\left(\frac{\partial {u}_{x}}{\partial x}+\frac{\partial {u}_{y}}{\partial y}\right)-{A}_{5}\frac{\partial {s}_{xy}}{\partial x}\\ {A}_{7}\frac{{\partial }^{2}{s}_{xy}}{\partial x\partial y}+2\left({2{A}_{0}+A}_{6}\right)\frac{\partial {u}_{x}}{\partial x}+2{A}_{6}\frac{\partial {u}_{y}}{\partial y}\\ {A}_{7}\frac{{\partial }^{2}{s}_{xy}}{\partial x\partial y}+2{A}_{6}\frac{\partial {u}_{x}}{\partial x}+2\left(2{A}_{0}+{A}_{6}\right)\frac{\partial {u}_{y}}{\partial y}\\ 2{A}_{0}\left(\frac{\partial {u}_{x}}{\partial y}+\frac{\partial {u}_{y}}{\partial x}\right)\end{array}\right).$$

Insertion of the coefficients of the matrix Eq. (33) and specifying the boundary conditions in COMSOL Multiphysics are performed considering the built-in tools in the software. The numerical solution is then carried out using the solver of the software while the convergence is achieved with sufficient number of triangular elements.

### The first boundary value problem

For numerical simulation in the first boundary value problem, we choose the following values for the applied displacement at the boundary.

34 $$u_{0} = - 0.05a,\quad \delta_{1} = 0.3a\quad \delta_{2} = \delta_{3} = \frac{a}{10}.$$

We also consider vanishing coupling parameters ${\mu }_{c}$ and ${\lambda }_{c}$ (i.e. ${\mu }_{c}={\lambda }_{c}=0$) while the length scale parameters take the values ${{\ell}}_{1}={{\ell}}_{2}=0.01, 0.004, 0.008, 0.001$.

**Figure 3:**
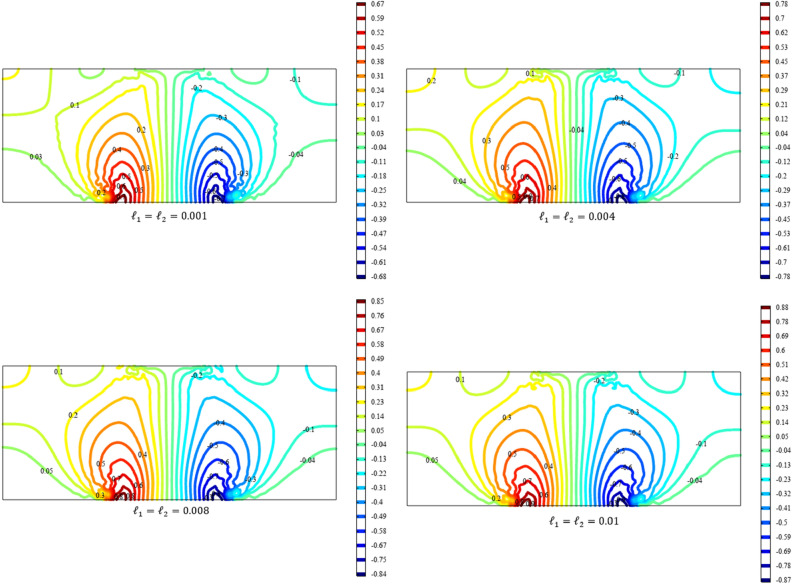
Contour plot for the displacement ${u}_{x}\left(x,y\right)/\left|{u}_{0}\right|$ for different values of ${{\ell}}_{1}$ and ${{\ell}}_{2}$ (COMSOL Multiphysics).

**Figure 4:**
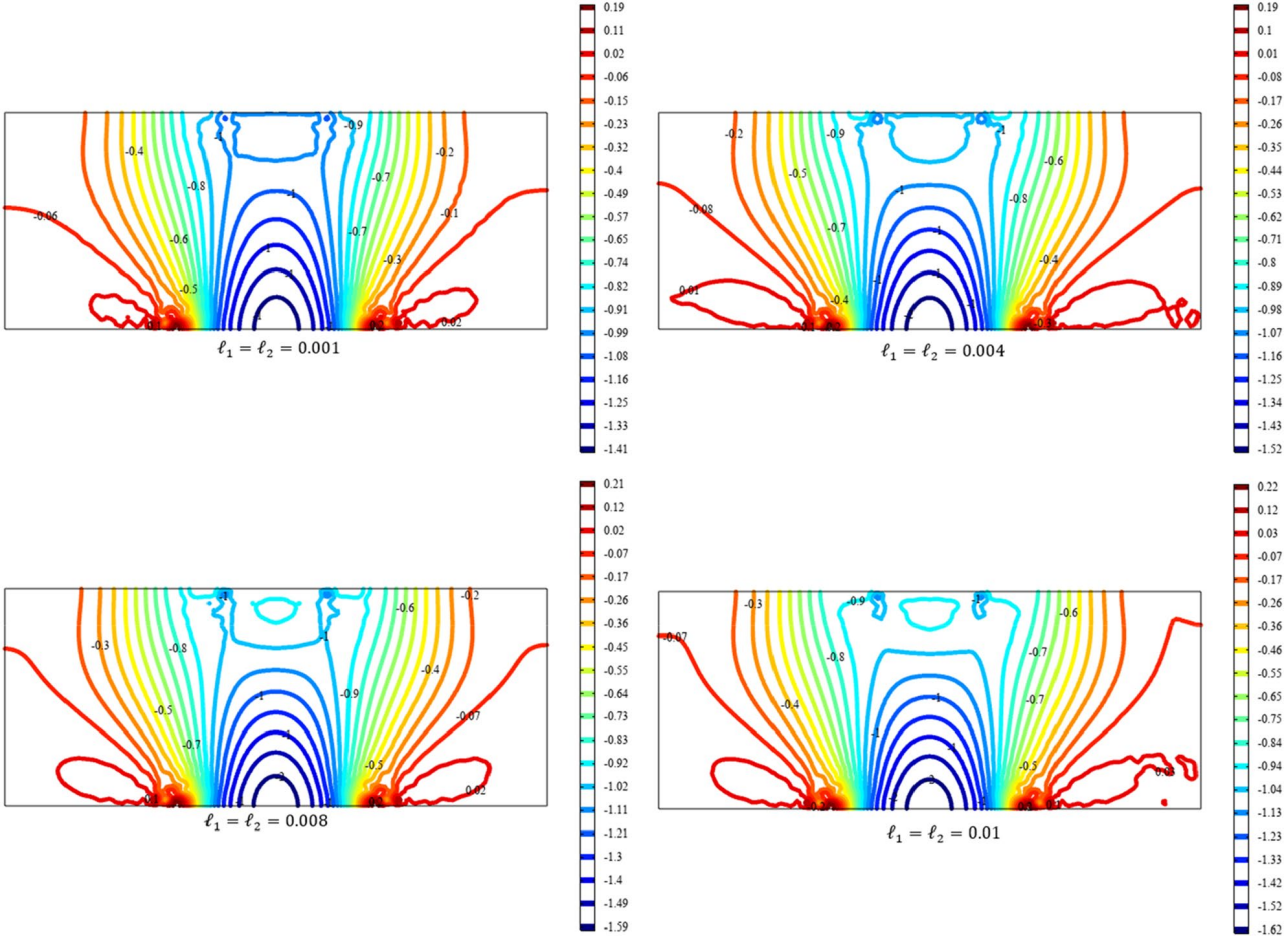
Contour plot for the displacement ${u}_{y}\left(x,y\right)/\left|{u}_{0}\right|$ for different values of ${{\ell}}_{1}$ and ${{\ell}}_{2}$(COMSOL Multiphysics).

**Figure 5:**
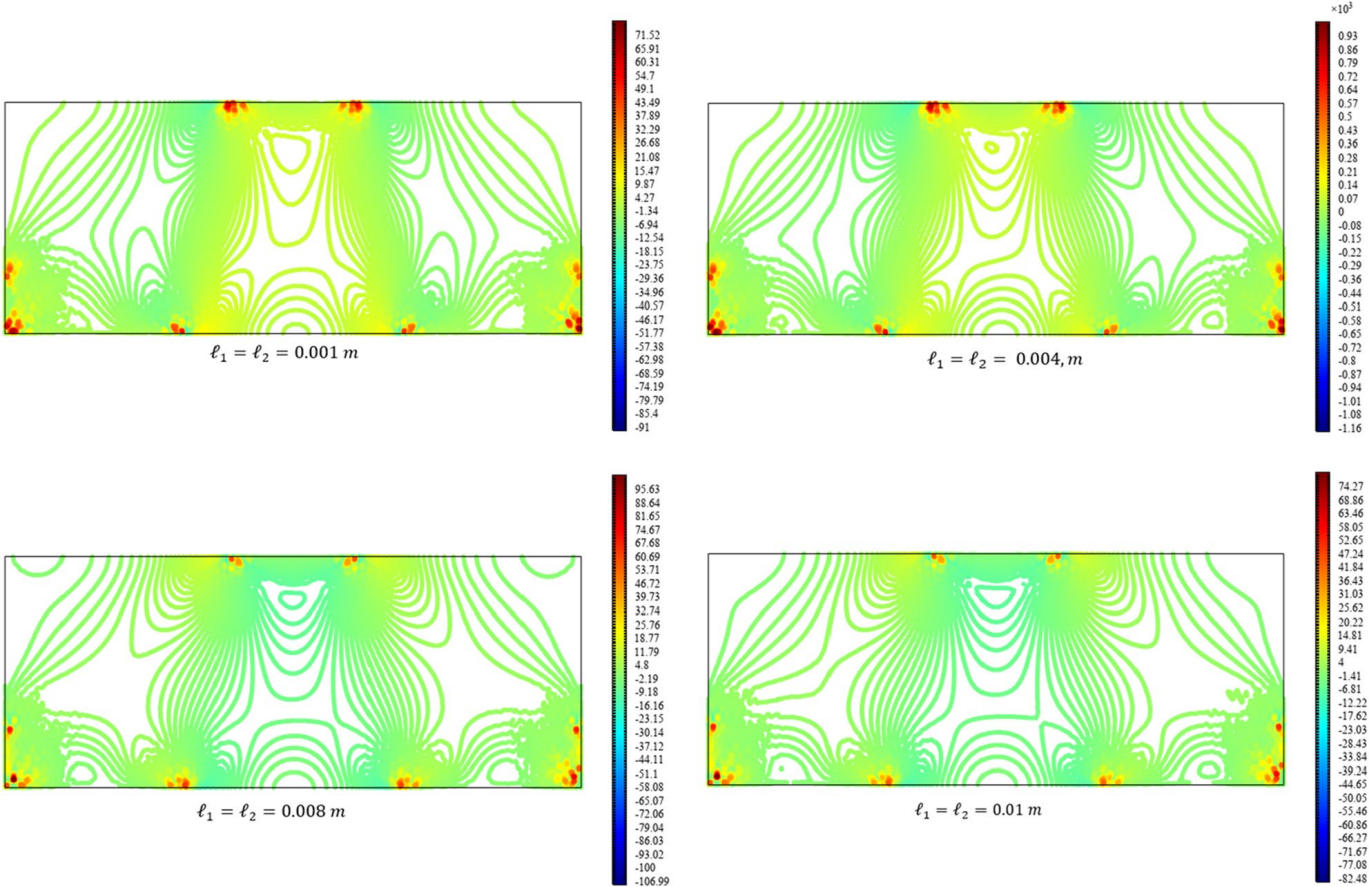
Contour plot for the micro-strain ${s}_{xx}\left(x,y\right)/{s}_{0}$ for different values of ${{\ell}}_{1}$ and ${{\ell}}_{2}$(COMSOL Multiphysics).

**Figure 6:**
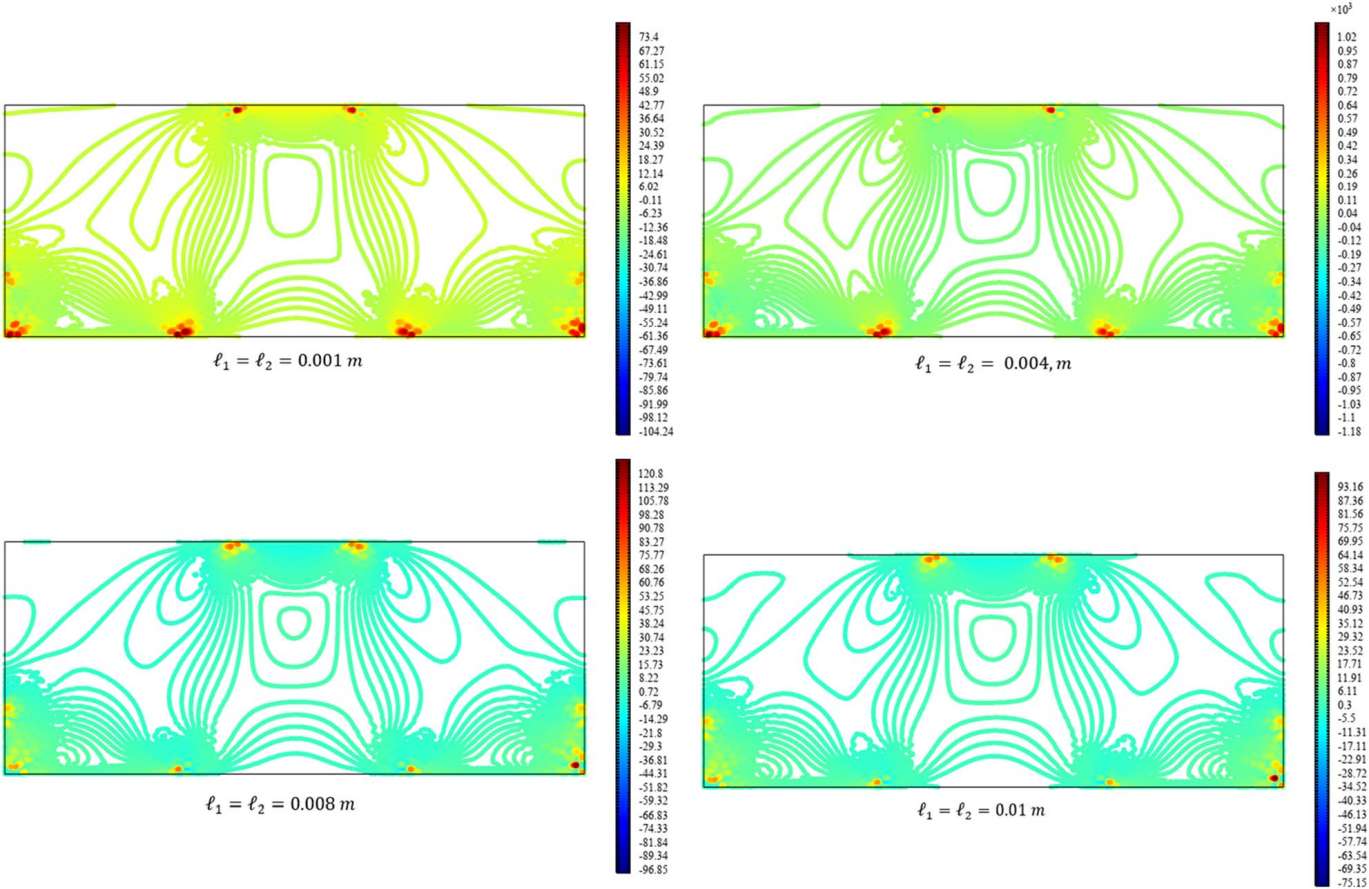
Contour plot for the micro-strain ${s}_{yy}\left(x,y\right)/{s}_{0}$ for different values of ${{\ell}}_{1}$ and ${{\ell}}_{2}$(COMSOL Multiphysics).

**Figure 7:**
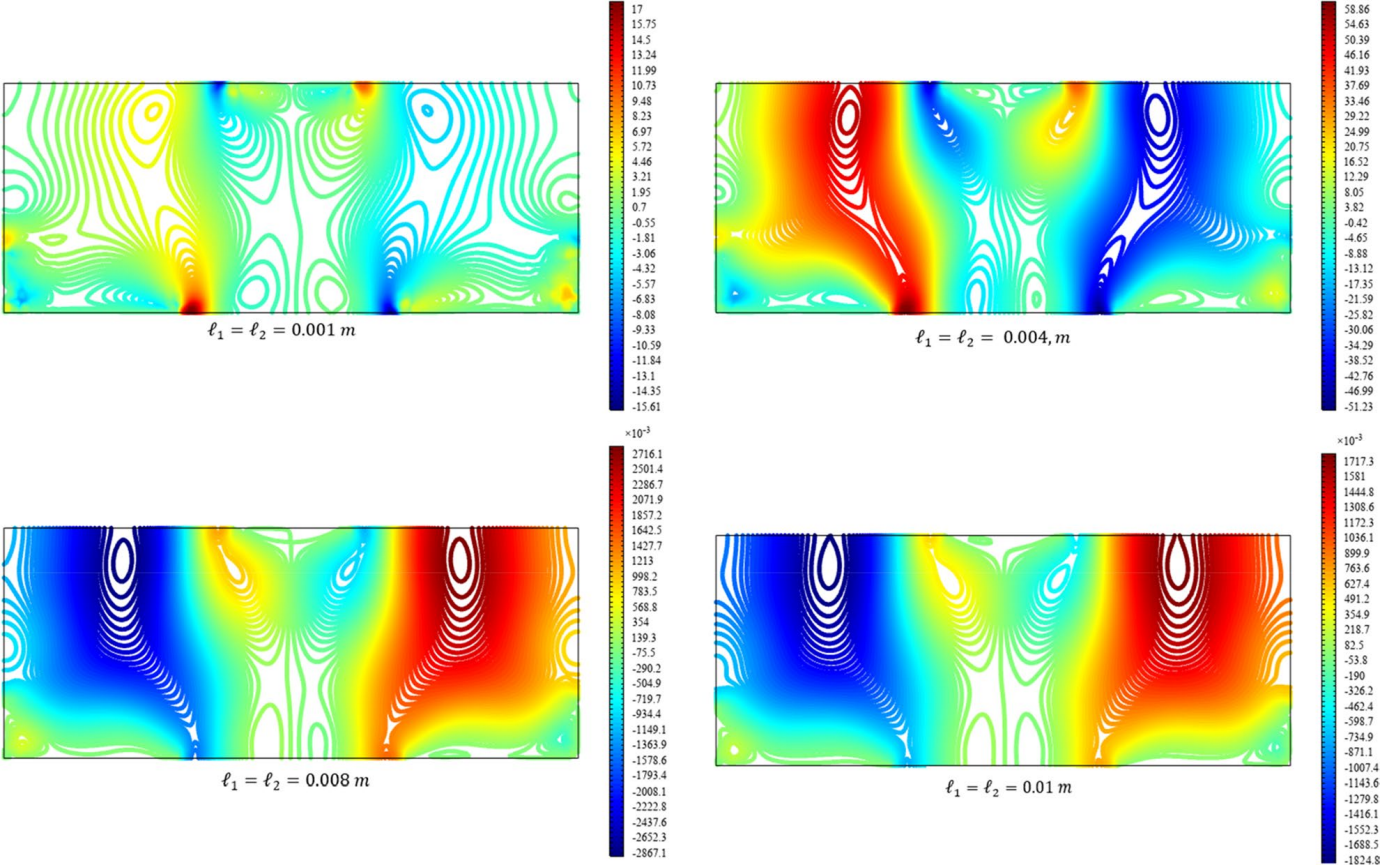
Surface plot for the micro-strain ${s}_{xy}\left(x,y\right)/{s}_{0}$ for different values of ${{\ell}}_{1}$ and ${{\ell}}_{2}$(COMSOL Multiphysics).

Figures 3, 4, 5, 6, 7 show the contour plot for the internal fields ${u}_{x}\left(x,y\right)$, ${u}_{y}\left(x,y\right)$, ${s}_{xx}\left(x,y\right)$, ${s}_{yy}\left(x,y\right)$, and ${s}_{xy}\left(x,y\right)$, respectively. The results are normalized with $\left|{u}_{0}\right|$ and ${s}_{0}=\sqrt{\frac{\mu }{{\mu }_{m}}}\frac{\left|{u}_{0}\right|}{b}$. The latter normalization is motivated by the form of the free energy. For colors in the figures, refer to the electronic version of the paper.

All boundary conditions, including both Neumann and Dirichlet conditions, are satisfied. The symmetry and anti-symmetry expected for the corresponding deformation measures are confirmed in the results presented in the figures. Interestingly, the ${s}_{xx}$ and ${s}_{yy}$ components of micro-strain appear only in the vicinity of the displaced boundaries while the ${s}_{xy}$ component of the micro-strain, similar to the displacements ${u}_{x}$ and ${u}_{y}$, is distributed over the entire domain.

Reducing the length scale parameters ${{\ell}}_{1}$ and ${{\ell}}_{1}$, as expected, eliminates the nonclassical measures i.e. micro-strains. It is noted that setting the length scale parameters ${{\ell}}_{1}$ and ${{\ell}}_{1}$ equal to zero cancels the effect of the gradient of the micro-strain. In this case, with vanishing ${\lambda }_{c}$ and ${\mu }_{c}$, the RMM reduces to the classical model of elasticity for which the numerical treatment developed here is to be reformulated and replaced by the finite element for classical elasticity.

**Figure 8:**
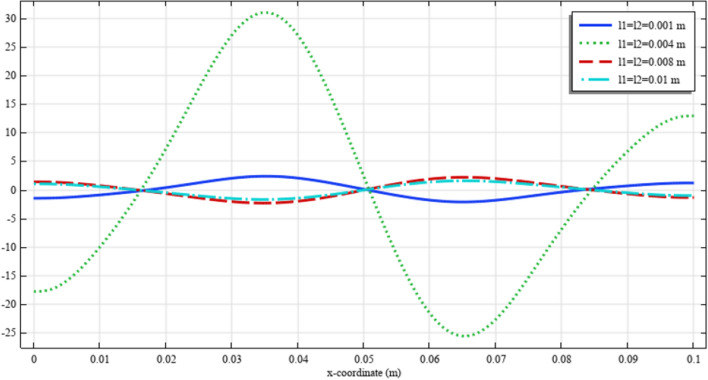
The displacement ${u}_{x}\left(x,b/2\right)/\left|{u}_{0}\right|$ for different values of ${{\ell}}_{1}$ and ${{\ell}}_{2}$(COMSOL Multiphysics).

**Figure 9:**
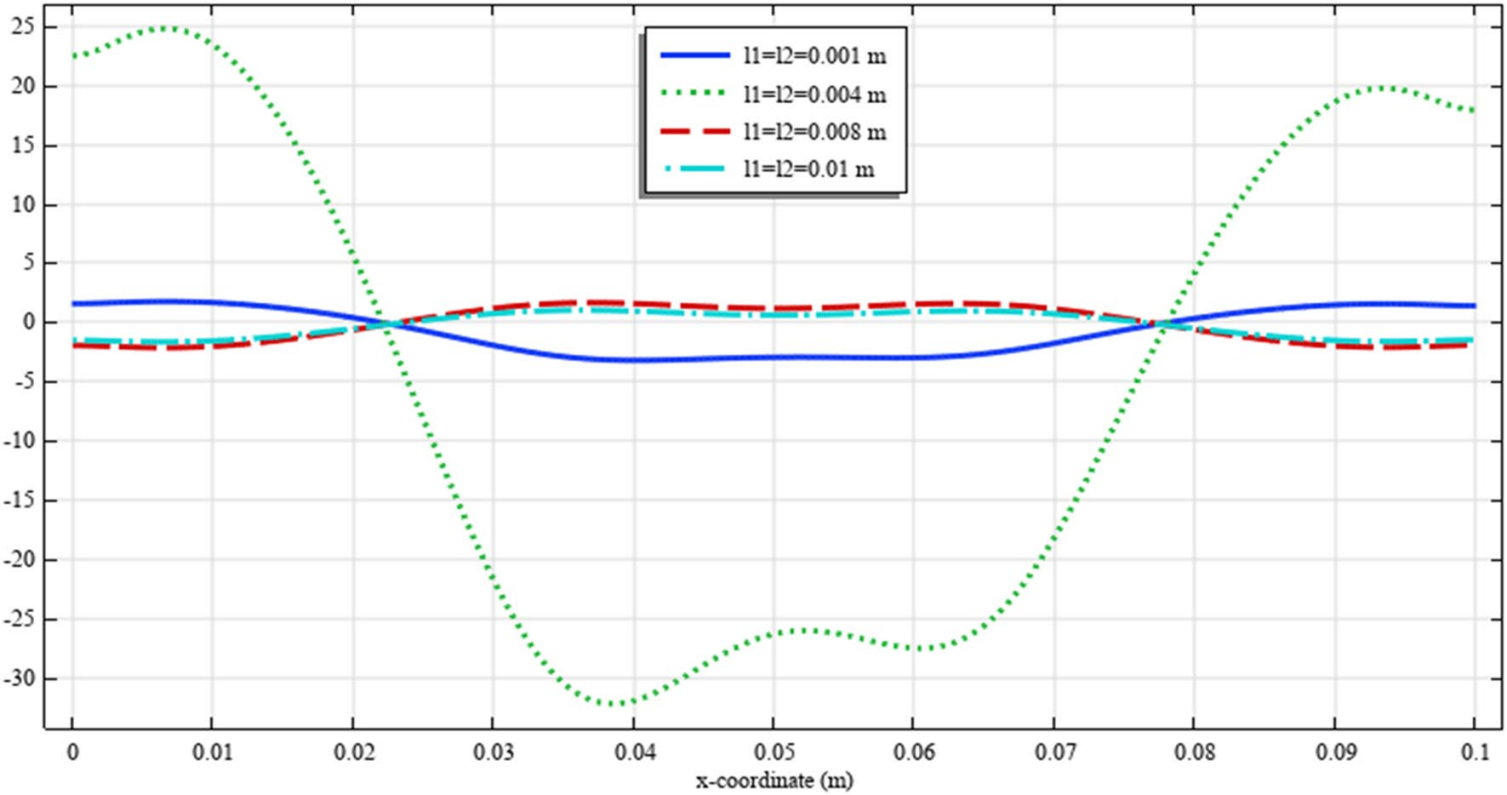
The displacement ${u}_{y}\left(x,b/2\right)/\left|{u}_{0}\right|$ for different values of ${{\ell}}_{1}$ and ${{\ell}}_{2}$(COMSOL Multiphysics).

**Figure 10:**
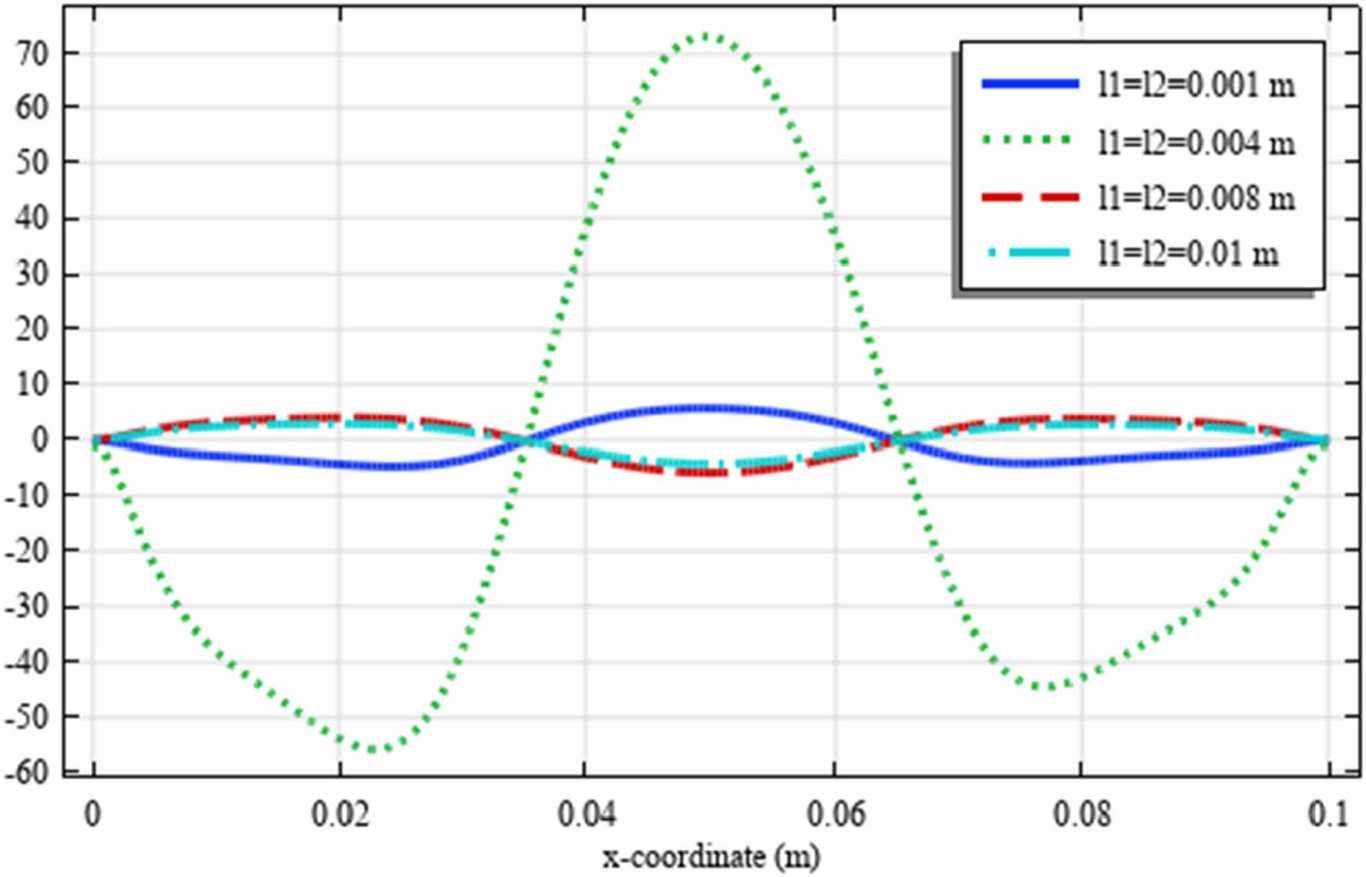
The micro-strain ${s}_{xx}\left(x,b/2\right)/{s}_{0}$ for different values of ${{\ell}}_{1}$ and ${{\ell}}_{2}$(COMSOL Multiphysics).

**Figure 11:**
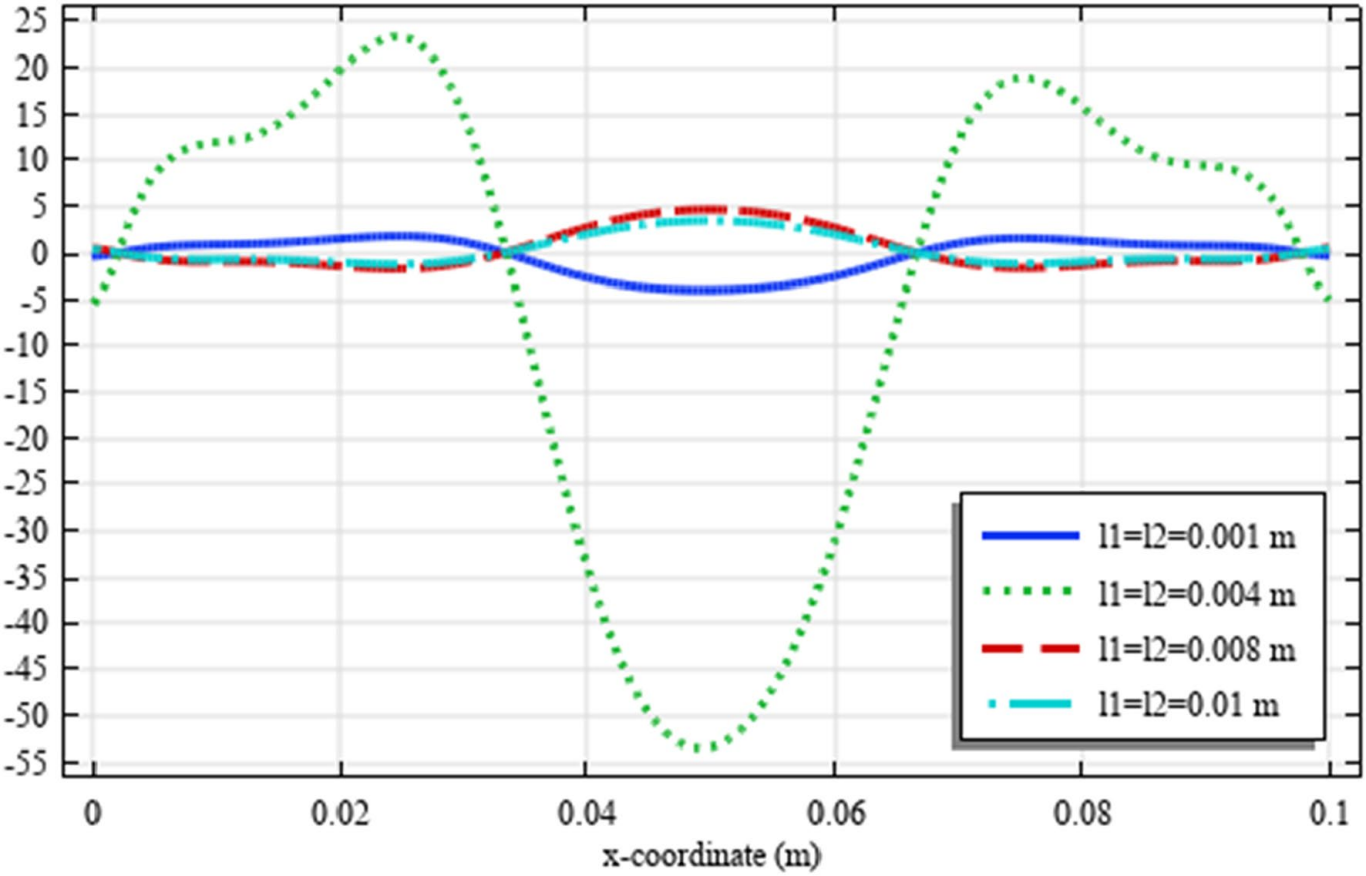
The micro-strain ${s}_{yy}\left(x,b/2\right)/{s}_{0}$ for different values of ${{\ell}}_{1}$ and ${{\ell}}_{2}$(COMSOL Multiphysics).

**Figure 12:**
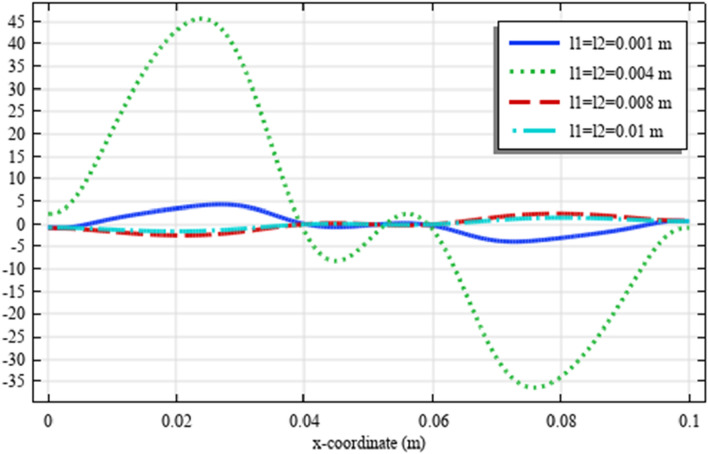
The micro-strain ${s}_{xy}\left(x,b/2\right)/{s}_{0}$ for different values of ${{\ell}}_{1}$ and ${{\ell}}_{2}$(COMSOL Multiphysics).

To elaborate the deformation pattern in the plane, the displacement and micro-strain components are demonstrated in Figs. 8, 9, 10, 11, 12 along the mid-line $y=b/2$. It is observed that the length scale parameter has a greater effect on micro-strain rather than the displacement components. It is also observed that the displacement component ${u}_{y}\left(x,b/2\right)$, the micro-strain component ${s}_{xx}\left(x,\mathrm{b}/2\right)$ and the micro-strain component ${s}_{yy}\left(x,\mathrm{b}/2\right)$ possess even symmetry about the central point $\left(0.05,b/2\right)$, while the other components depict odd symmetry.

### The second boundary value problem

For the numerical simulation of the second boundary value problem, the following values are selected for the applied displacement at the boundary.

35 $$u_{0} = - 0.05a,\quad { }\delta_{1} = 0.2a{ }, \quad \delta_{2} = 0.4a.$$

Considering these values together with ${\mu }_{c}={\lambda }_{c}=0, {{\ell}}_{1}={{\ell}}_{2}=0, 0.001, 0.004, 0.008, 0.01 m$, the internal fields of the RMM plane are obtained.

**Figure 13:**
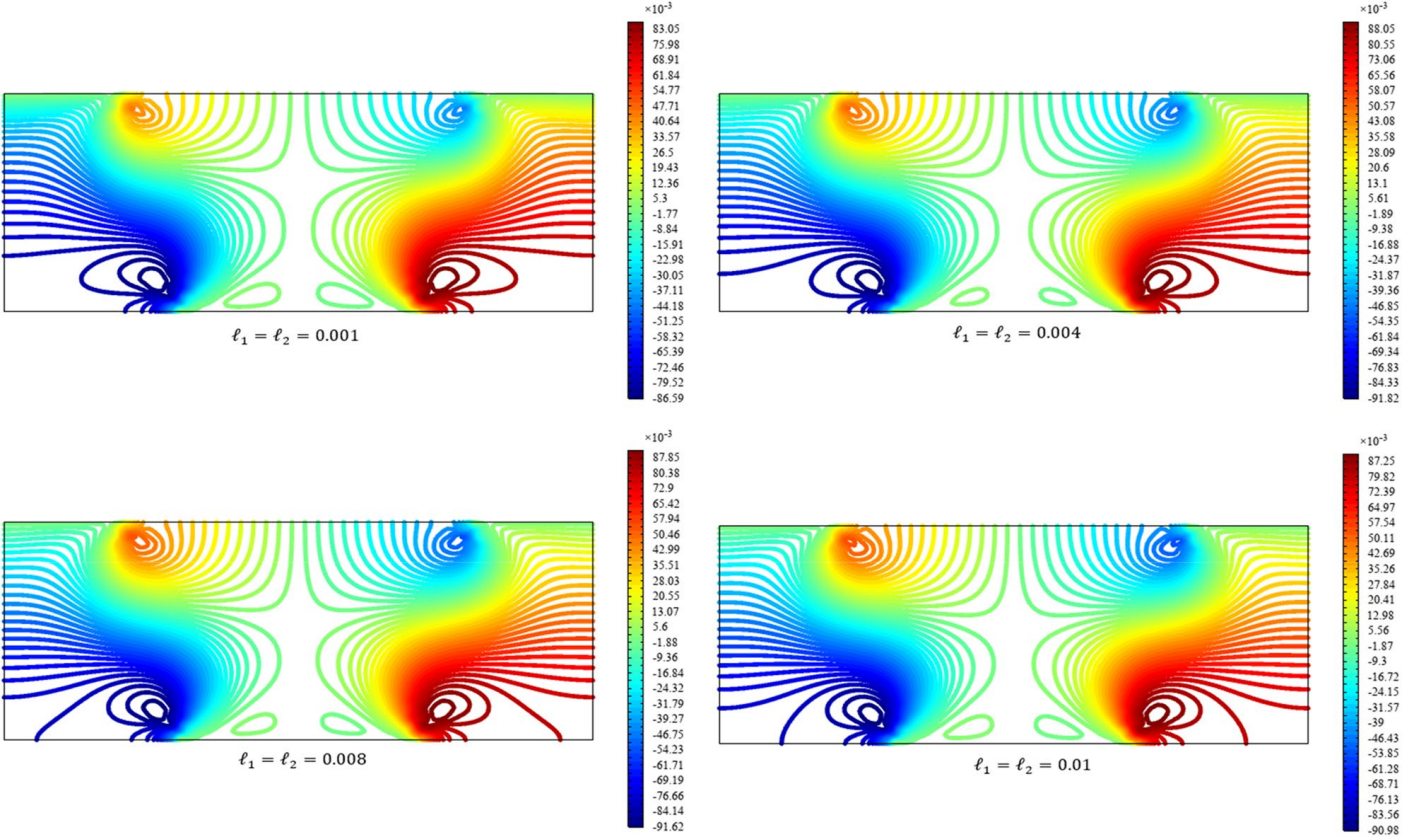
Contour plot for the displacement ${u}_{x}\left(x,y\right)/\left|{u}_{0}\right|$ for different values of ${{\ell}}_{1}$ and ${{\ell}}_{2}$(COMSOL Multiphysics).

**Figure 14:**
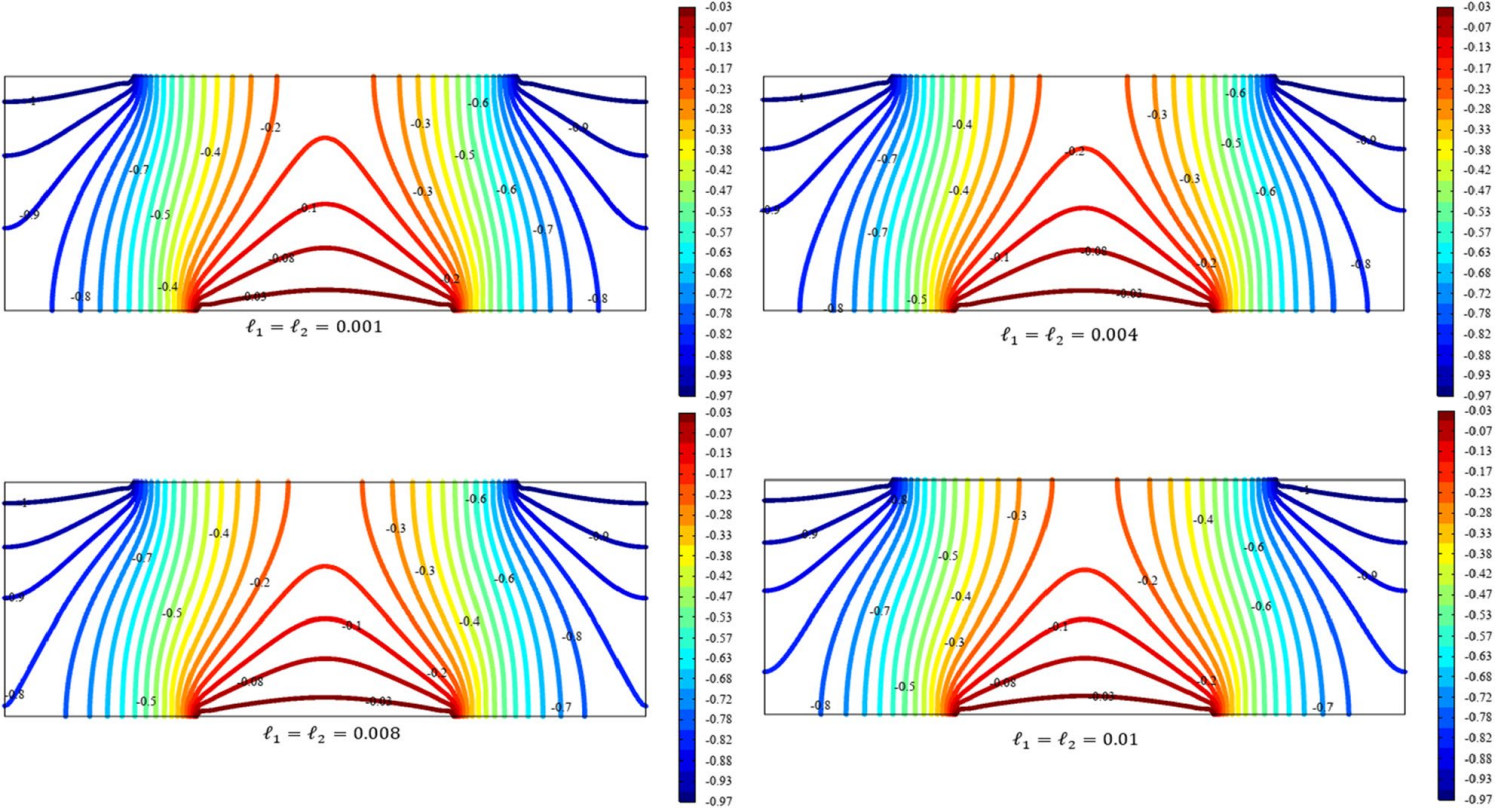
Contour plot for the displacement ${u}_{y}\left(x,y\right)/\left|{u}_{0}\right|$ for different values of ${{\ell}}_{1}$ and ${{\ell}}_{2}$(COMSOL Multiphysics).

**Figure 15:**
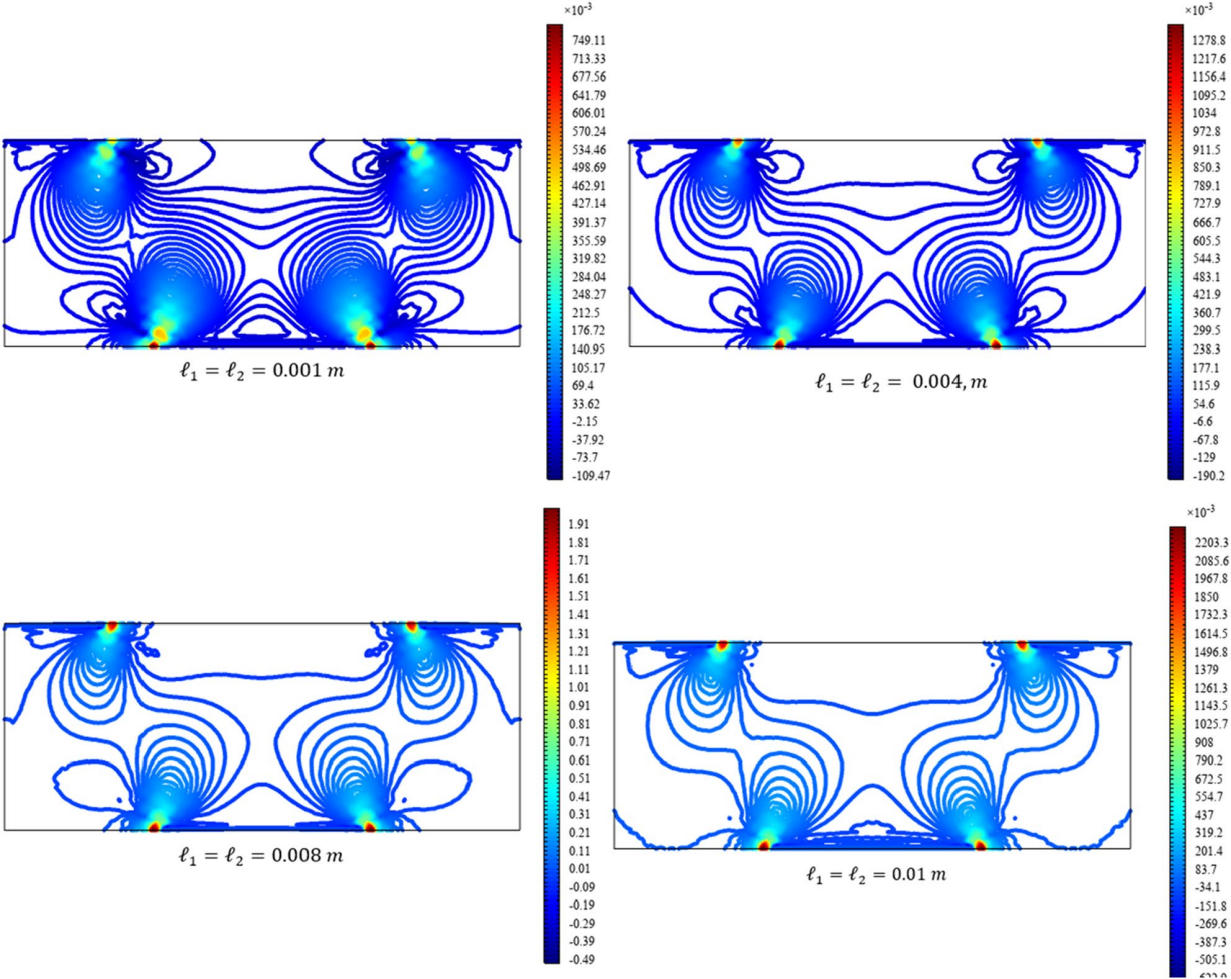
Contour plot for the micro-strain ${s}_{xx}\left(x,y\right)/\left|{u}_{0}\right|$ for different values of ${{\ell}}_{1}$ and ${{\ell}}_{2}$(COMSOL Multiphysics).

**Figure 16:**
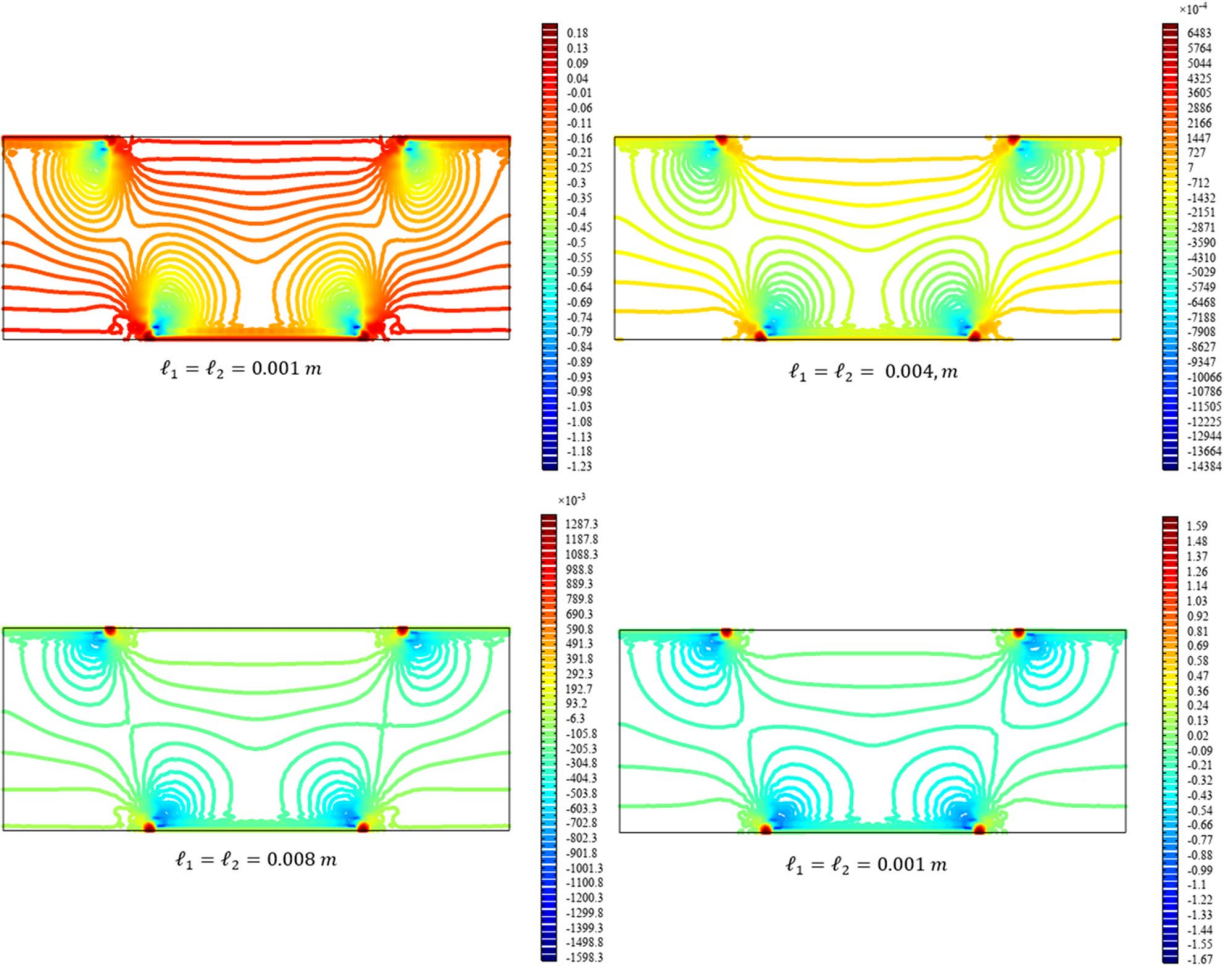
Contour plot for the micro-strain ${s}_{yy}\left(x,y\right)/{s}_{0}$ for different values of ${{\ell}}_{1}$ and ${{\ell}}_{2}$(COMSOL Multiphysics).

**Figure 17:**
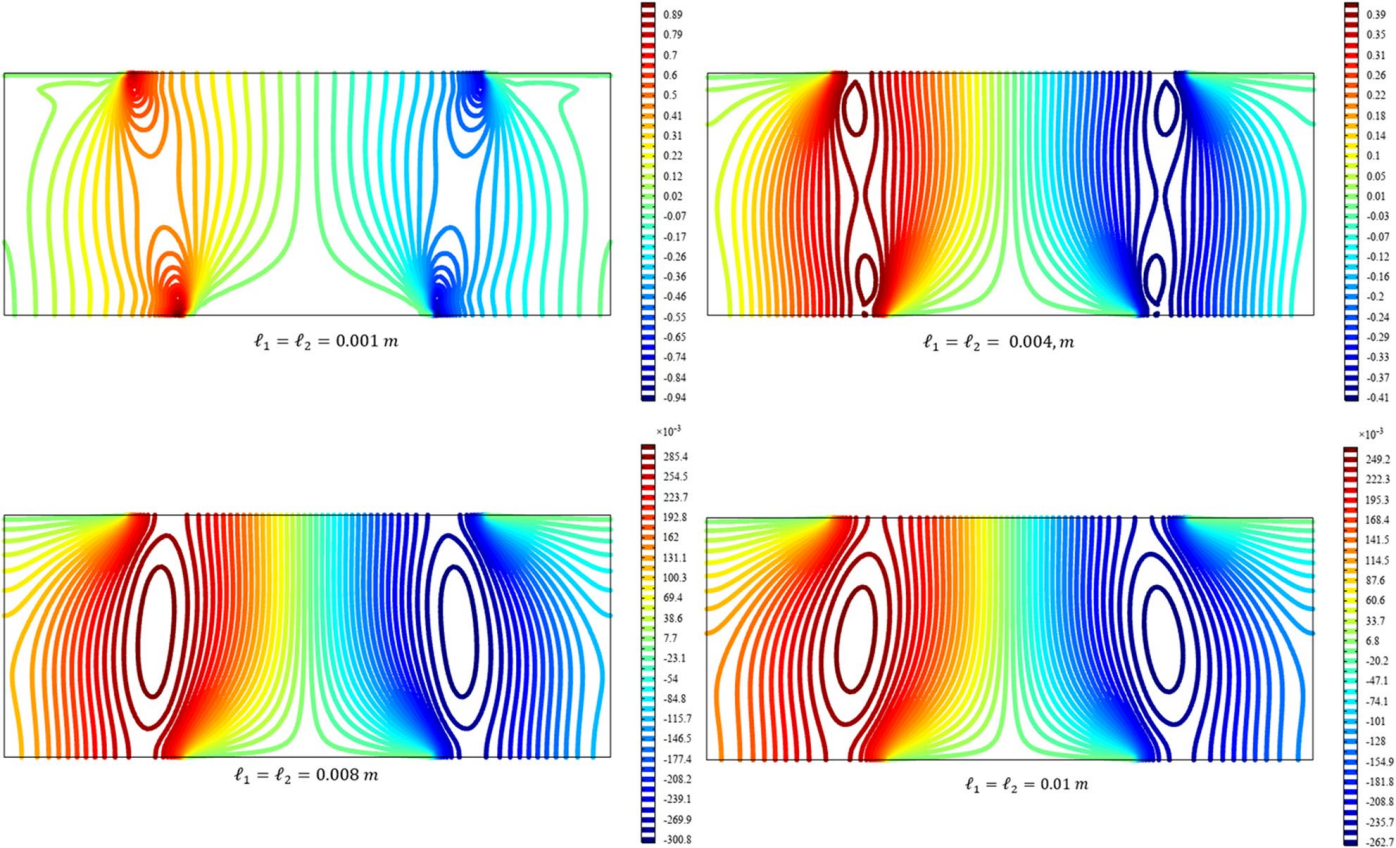
Contour plot for the micro-strain ${s}_{xy}\left(x,y\right)/{s}_{0}$ for different values of ${{\ell}}_{1}$ and ${{\ell}}_{2}$(COMSOL Multiphysics).

Figures 13, 14, 15, 16, 17 show the contour plot for the internal fields ${u}_{x}\left(x,y\right)$, ${u}_{y}\left(x,y\right)$, ${s}_{xx}\left(x,y\right)$, ${s}_{yy}\left(x,y\right)$, and ${s}_{xy}\left(x,y\right)$, respectively. Similar to the first BVP, the micro-strain vanishes when reducing the length scale parameters *l*_1_ and *l*_2_. Moreover, the ${s}_{xx}$ and ${s}_{yy}$ components of micro-strain only appears in the neighborhood of the external excitation while the ${s}_{xy}$ component of micro-strain is distributed over the entire domain.

**Figure 18:**
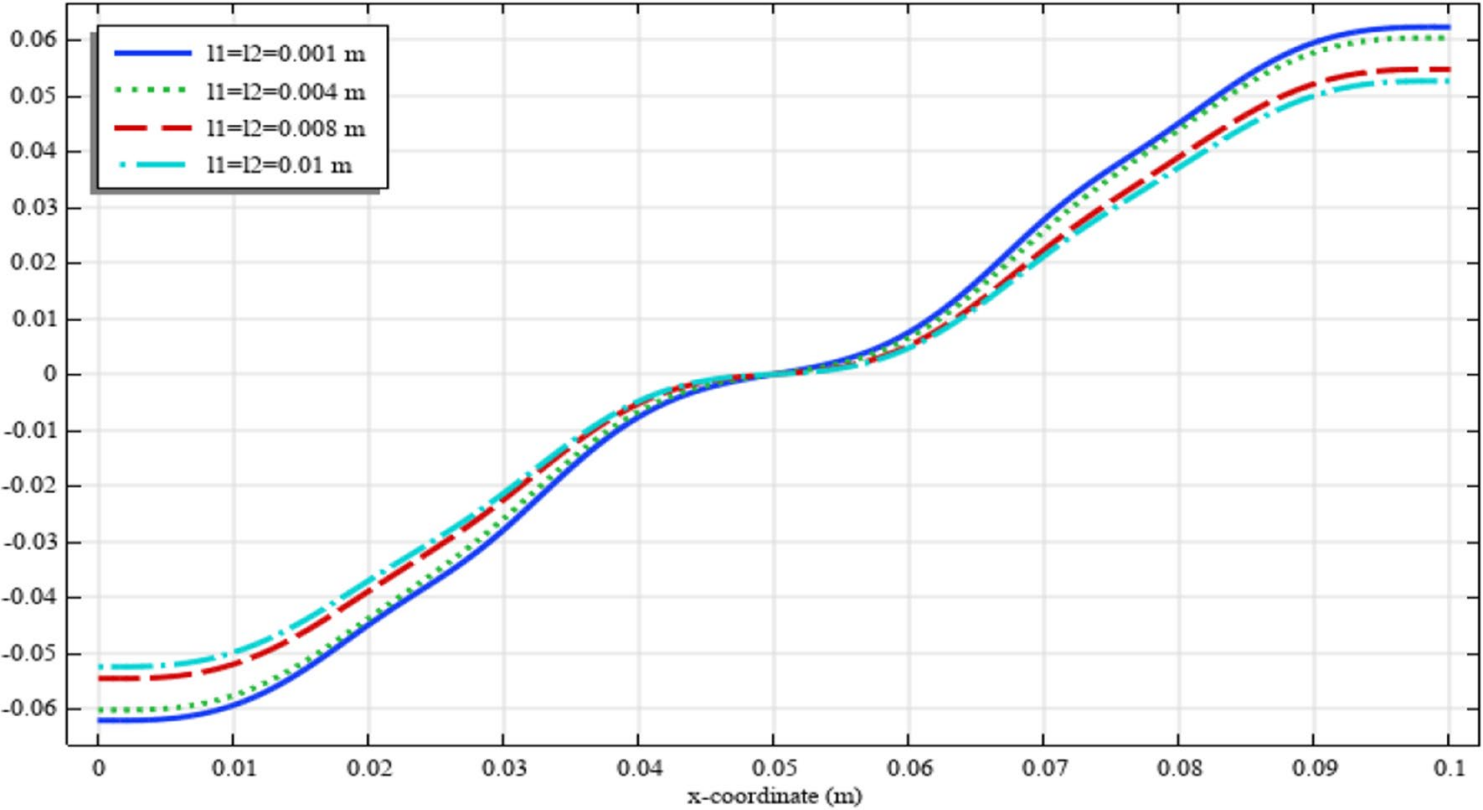
The displacement ${u}_{x}\left(x,b/2\right)/\left|{u}_{0}\right|$ for different values of ${{\ell}}_{1}$ and ${{\ell}}_{2}$(COMSOL Multiphysics).

**Figure 19:**
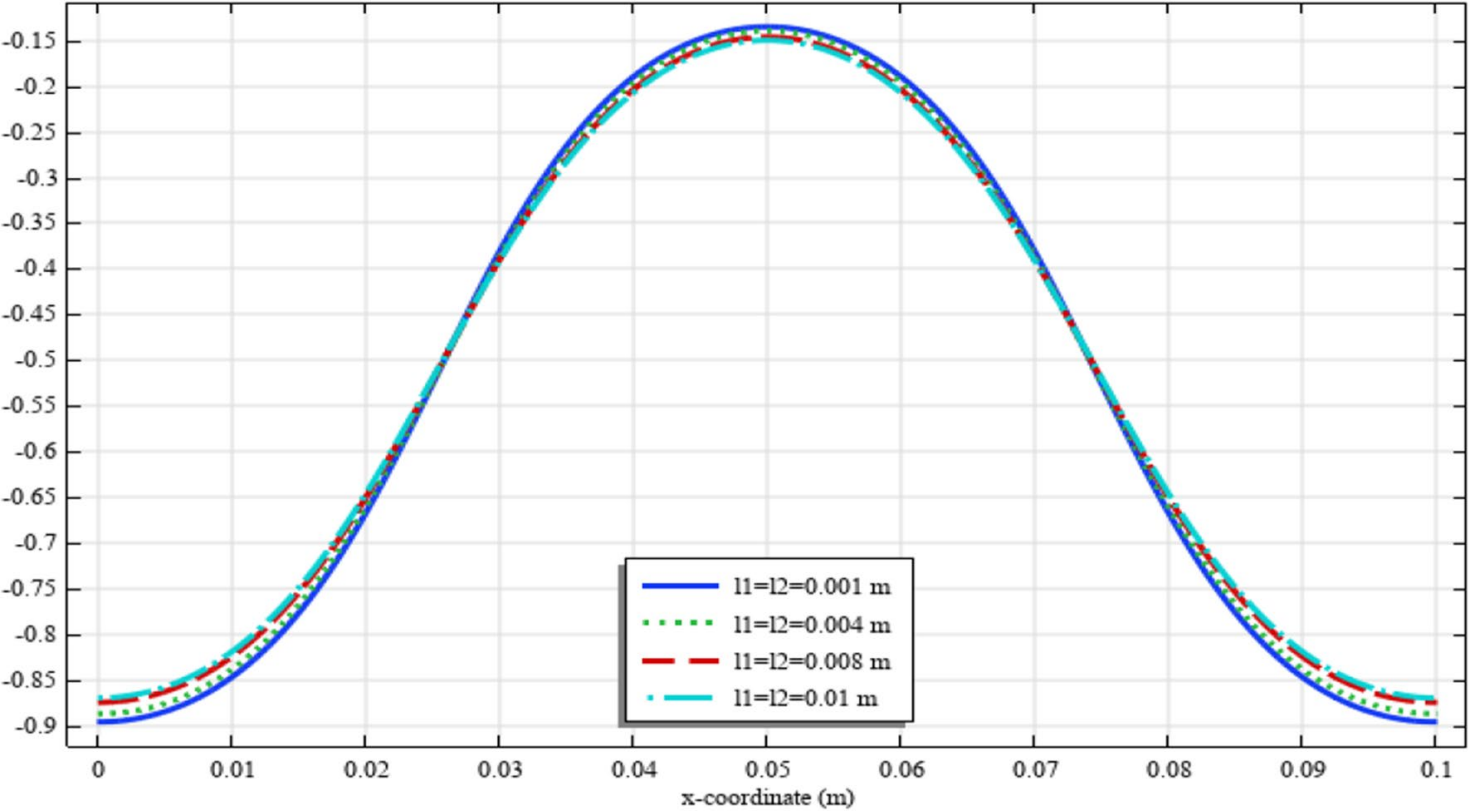
The displacement ${u}_{y}\left(x,b/2\right)/\left|{u}_{0}\right|$ for different values of ${{\ell}}_{1}$ and ${{\ell}}_{2}$(COMSOL Multiphysics).

**Figure 20:**
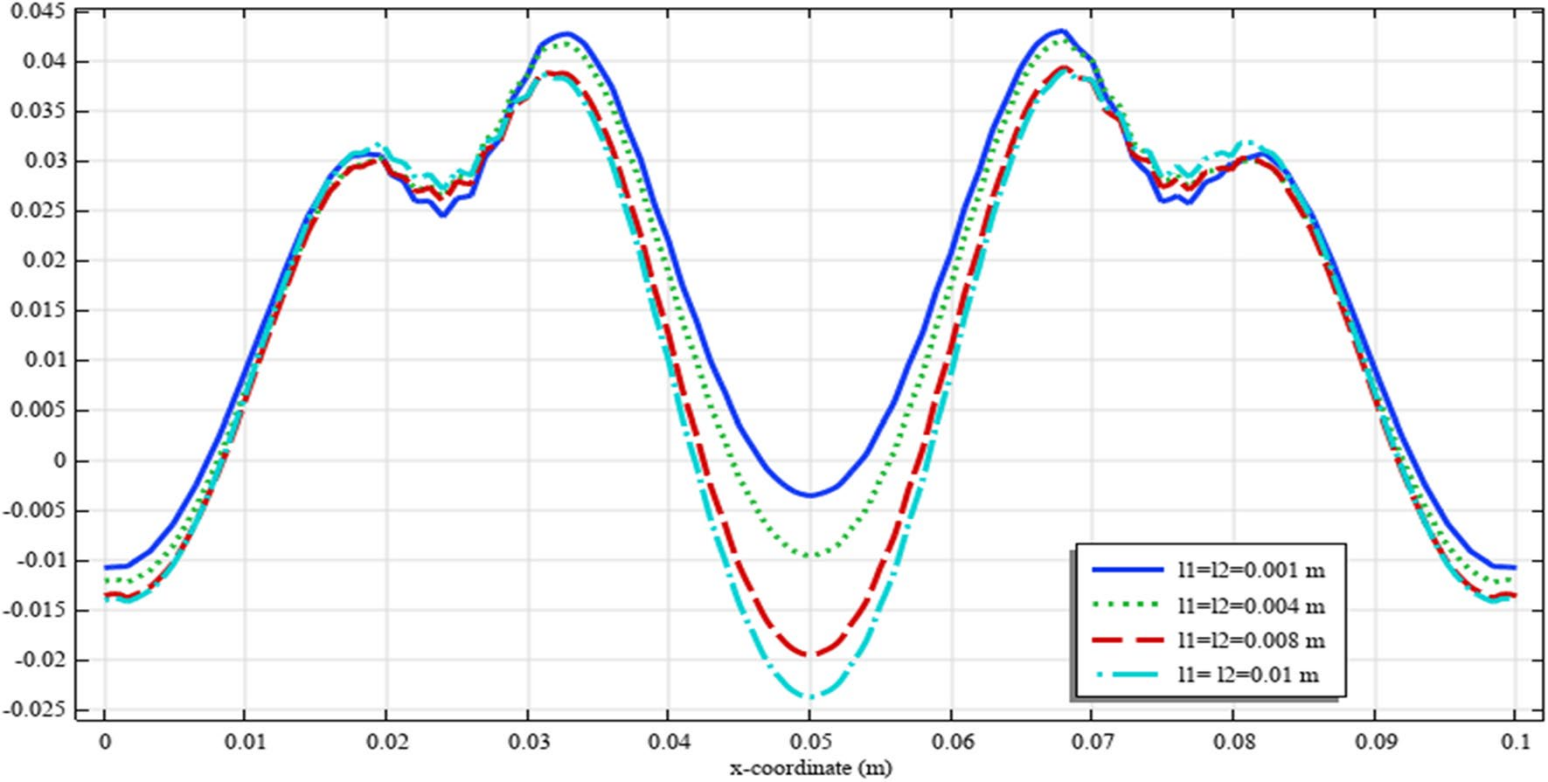
The micro-strain ${s}_{xx}\left(x,b/2\right)/{s}_{0}$ for different values of ${{\ell}}_{1}$ and ${{\ell}}_{2}$(COMSOL Multiphysics).

**Figure 21:**
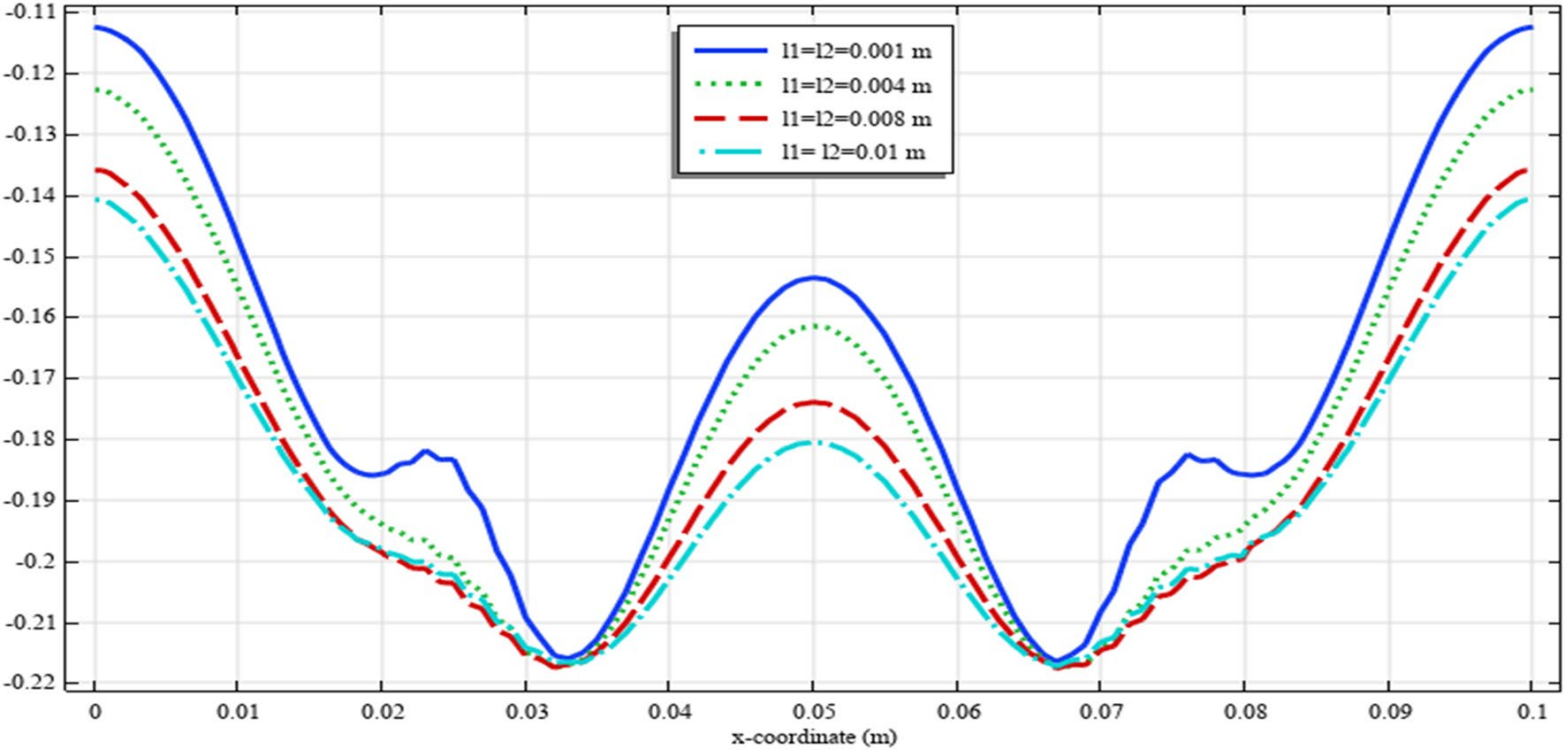
The micro-strain ${s}_{yy}\left(x,b/2\right)/{s}_{0}$. for different values of $\ell_{1}$ and $\ell_{2}$(COMSOL Multiphysics).

**Figure 22:**
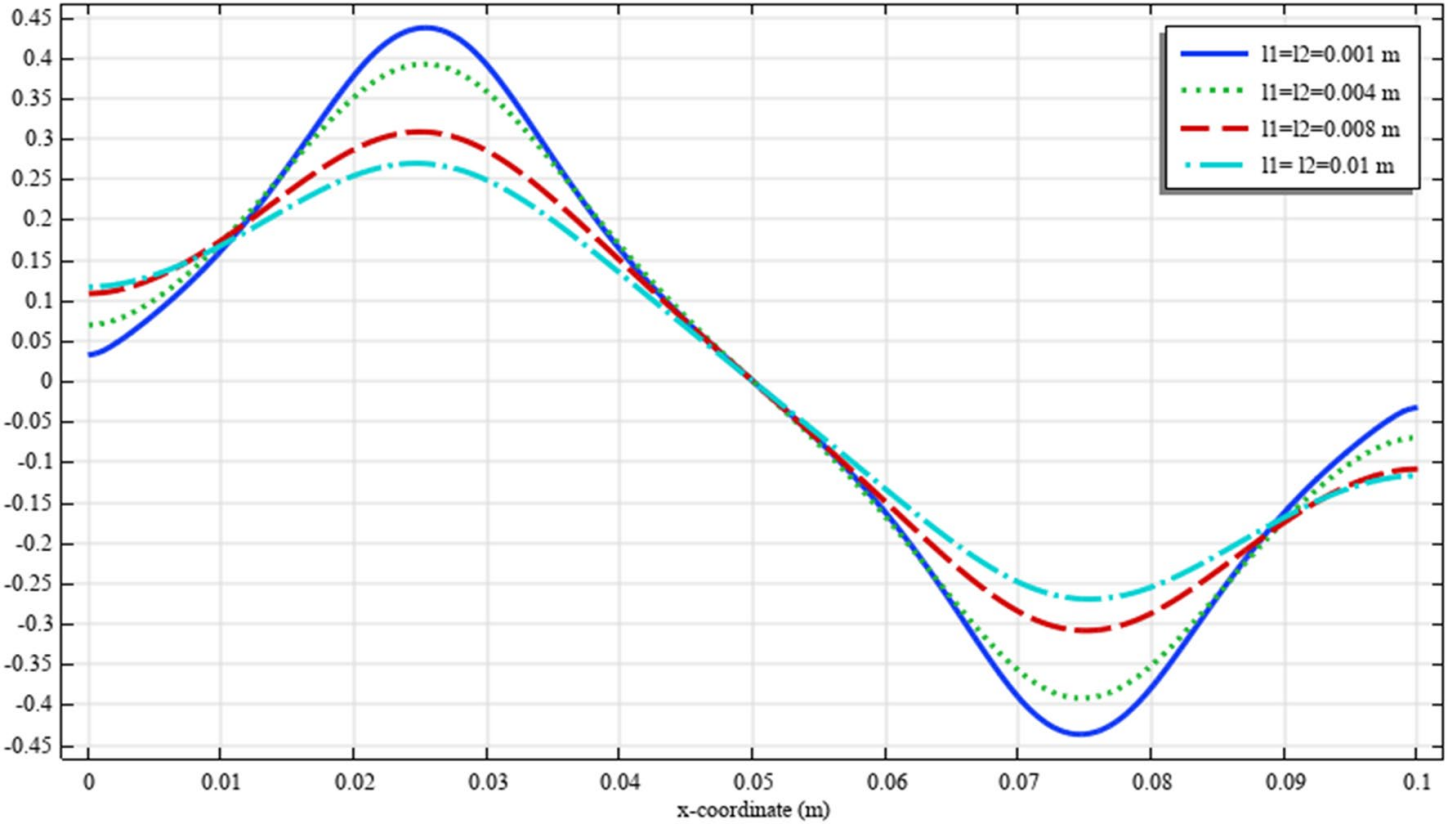
The micro-strain $s_{xy} \left( {x,b/2} \right)/s_{0}$ for different values of $\ell_{1}$ and $\ell_{2}$(COMSOL Multiphysics).

Figures 18, 19, 20, 21, 22 show the displacement and micro-strain fields of the homogenized domain along the midline $y=b/2$ with changing the parameter ${{\ell}}_{1}$ and ${{\ell}}_{2}$ while ${\mu }_{c}={\lambda }_{c}=0$. It is again noticed that the nonclassical quantities, i.e. micro-strains, are considerably affected with the variation of the length scales.

## Conclusion

In this article, the reduced micromorphic model is used for the static analysis of a plane. The variational method is used to get the field equations and boundary conditions for the proposed quantities. The main difference between the present model and the classical model is the set of the deformation measures including classical strain tensor, micro-strain tensor, and residual strain tensor. One of the proposed quantities, micro-strain tensor, is used as a measure of the internal interaction of microelements due to the externally applied fields.

The governing equations and the specified boundary conditions were introduced in Cartesian coordinates to describe a rectangular domain. The numerical solution based on the finite element method were derived using the COMSOL Multiphysics. In order to elaborate the material behavior in the reduced micromorphic model, two case studies were discussed. The first case was a rectangular plane possessing one displaced boundary and two supports while the second case was a rectangular plane with two displaced boundaries and one support. The results are illustrated graphically and discussed. The most admissible results are that the micro-strain tensor is concentrated around the displaced boundary and supports while the displacement is distributed uniformly over the entire domain of the solution.

In future studies, specific microstructures/microarchitectures will be considered and homogenized computationally towards reduced micromorphic model. The deformation patterns obtained in this study can be used for the identification of the microarchitectures whose behavior can be captured by reduced micromorphic model. Furthermore, in the context of laboratory experiments and based on digital image correlation, the results of this study (in particular the contours of the field quantities) can also guide for identifying the heterogenous materials whose behavior can be well described with reduced micromorphic model.

## Abbreviations

$$u_{i} , i = 1,2,3$$: Displacement components
$\varepsilon_{ij} = \left( {u_{i,j} + u_{j,i} } \right)/2 = \varepsilon_{ji}$: Classical strain tensor
$s_{ij} = s_{ji} , i,j = 1,2,3$: Micro-strain tensor
$t_{ij} = t_{ji} , i,j = 1,2,3$: Micro-stress tensor
$\tau_{ij} = \tau_{ji} , i,j = 1,2,3$: Cauchy-like tensor or residual stress tensor
$m_{ijk} = m_{ikj}\,\, i,j,k = 1,2,3$: Higher order stress tensor
$\rho$ and $\rho_{m}$: Macroscopic mass density and mass density of a material particle
$$J$$: Micro-inertia
$$f_{i} , i = 1,2,3$$: Body force
$H_{jk} , j,k = 1,2,3$: Body higher-order moment
$\overline{t}_{i} , i = 1,2,3$: Cauchy stress vector
$\overline{m}_{jk} , j,k = 1,2,3$: Higher stress tensor
$F_{i}^{ex} , i = 1,2,3$: Wedge forces at the corners
$\lambda_{m}$ and $\mu_{m}$: Elastic moduli of the microstructure,
$\lambda$ and $\mu$: Elastic moduli of the matrix material.
$\lambda_{c}$ and $\mu_{c}$: Elastic moduli for coupling between micro-strain and macro-strain
$\ell_{1}$ and $\ell_{2}$: Length scale parameters
$a$ and $b$: Height and width of the rectangle domain
